# Isomer Information from Ion Mobility Separation of High-Mannose Glycan Fragments

**DOI:** 10.1007/s13361-018-1890-5

**Published:** 2018-03-05

**Authors:** David J. Harvey, Gemma E. Seabright, Snezana Vasiljevic, Max Crispin, Weston B. Struwe

**Affiliations:** 1 Target Discovery Institute, Nuffield Department of Medicine, University of Oxford, Roosevelt Drive, Oxford, OX3 7FZ UK; 2 Center for Biological Sciences, Faculty of Natural and Environmental Sciences, Life Sciences Building 85, University of Southampton, Highfield Campus, Southampton, SO17 1BJ UK; 3 Department of Biochemistry, University of Oxford, South Parks Road, Oxford, OX1 3QU UK; 4 Department of Chemistry, Chemistry Research Laboratory, University of Oxford, 12 Mansfield Road, Oxford, OX1 3TA UK

**Keywords:** Traveling wave, Ion mobility, Collision cross-sections, High-mannose *N*-glycans, Fragment ions

## Abstract

Extracted arrival time distributions of negative ion CID-derived fragments produced prior to traveling-wave ion mobility separation were evaluated for their ability to provide structural information on *N*-linked glycans. Fragmentation of high-mannose glycans released from several glycoproteins, including those from viral sources, provided over 50 fragments, many of which gave unique collisional cross-sections and provided additional information used to assign structural isomers. For example, cross-ring fragments arising from cleavage of the reducing terminal GlcNAc residue on Man_8_GlcNAc_2_ isomers have unique collision cross-sections enabling isomers to be differentiated in mixtures. Specific fragment collision cross-sections enabled identification of glycans, the antennae of which terminated in the antigenic α-galactose residue, and ions defining the composition of the 6-antenna of several of the glycans were also found to have different cross-sections from isomeric ions produced in the same spectra. Potential mechanisms for the formation of the various ions are discussed and the estimated collisional cross-sections are tabulated.

**Electronic supplementary material:**

The online version of this article (10.1007/s13361-018-1890-5) contains supplementary material, which is available to authorized users.

**Graphical Abstract:**
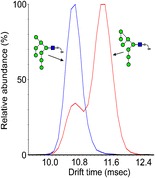
ᅟ

## Introduction

**Scheme 1:**
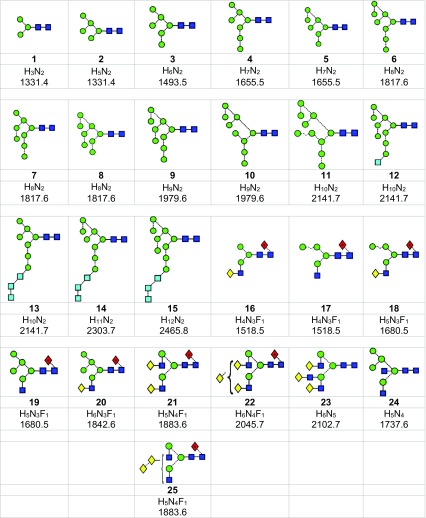
Structures of the *N*-glycans discussed in this paper. Symbols show the constituent monosaccharides using the scheme devised by Harvey et al. [77].  = GlcNAc,  = mannose,  = galactose,  = glucose,  = fucose. Colours are those recommended by the Consortium for Functional Glycomics (CFG). The angles of the bonds linking the symbols denote the linkages between the monosaccharide residues: | = 2-link, / = 3-link, - = 4-link, \ = 6=link. Full lines are β-bonds, broken lines are α-bonds. Masses are those of the [M+H_2_PO_4_]^-^ ions

**Figure 1:**
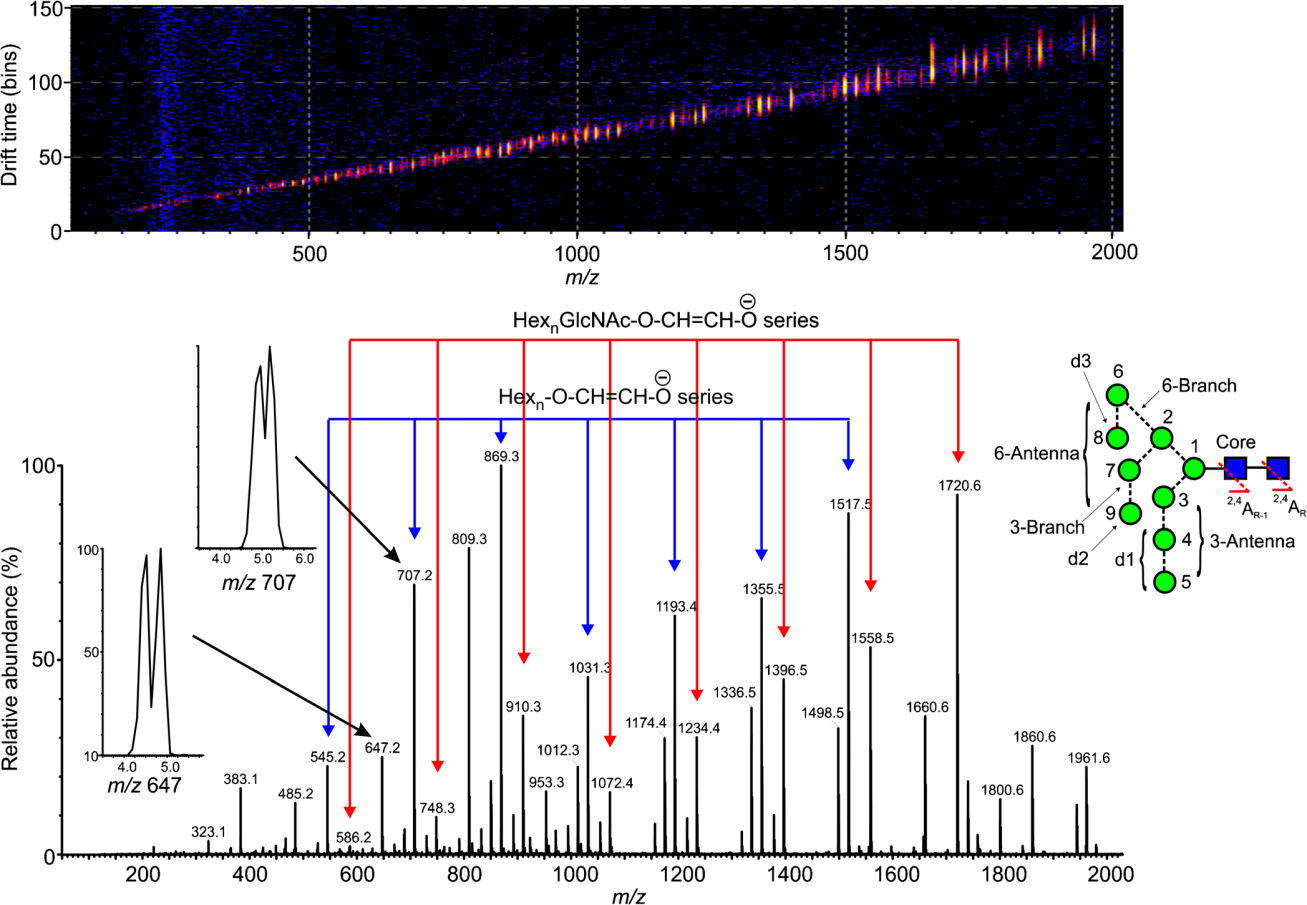
(**top**) Driftplot display of the fragment ions from Man_9_GlcNAc_2_ (**9**) produced in the trap region of the Synapt G2Si mass spectrometer. The spectrum of this compound is shown below with the two main series of ions discussed in this paper (Hex_n_-O-CH=CH-O^-^ (red) and Hex_n_GlcNAc-O-CH=CH-O^-^, blue) indicated. The two inserts show separation of two isomeric structures for *m/z* 647 and *m/z* 707. To the left is the structure of Man_9_GlcNAc_2_ showing the numbering system and the various regions of the molecule discussed in the text

N-Glycans are those carbohydrates attached to asparagine residues [1] in about half of the known proteins and are critical for many of the properties of these compounds, such as cell-cell adhesion and half-life. All contain a chitobiose core attached to three mannose residues to which several glycan chains called antennae are attached. They are biosynthesised [2] in most species, including mammals, by attachment of the glycan Glc_3_Man_9_GlcNAc_2_ (**15**, Scheme 1) to the nascent protein followed by removal of the glucose and α-mannose residues to give Man_5_GlcNAc_2_ (**2**). These compounds and the intermediate glycans such as **3**-**14** are known as high-mannose glycans. Addition of GlcNAc and galactose residues to the 3-mannose residue (the nomenclature for describing features of the high-mannose glycans is outlined in Figure 1) yields so-called hybrid glycans **16**-**20**) and removal of the 6 and 7 mannose residues from these hybrid glycans, followed by similar attachment of GlcNAc and galactose residues gives compounds known as complex glycans (e.g., the biantennary glycans **21** and **22**). Furthermore, these glycans can be decorated with further glycan residues such as *N*-acetylneuraminic (sialic) acid and fucose (**17**-**22**) at various positions.

The high-mannose glycans are now key targets in vaccine design such as those against the human immunodeficiency virus (HIV) [3, 4] where the sole target for antibody neutralisation is the glycoprotein, Env, a heavily glycosylated glycoprotein consisting of a trimer of gp120/gp41 heterodimers. Understanding the detailed structure of the high-mannose glycans, several of which occur as isomers, has prompted increased interest in methods for their analysis.

Structural analysis of these compounds, following their release from the protein, can be accomplished with various techniques, of which mass spectrometry plays a key role. Ion mobility has recently been shown to provide another dimension to the mass spectrometric analysis of carbohydrates (for recent reviews, see [5–7]) by, for example, its ability to separate isomers [8–41] and to enable glycan ions to be selectively extracted from complex and sometimes contaminated samples [42–47]. Combined with negative ion fragmentation [48–51] of native (underivatized) glycans, this technique is proving to be an ideal method for the analysis of *N*-linked glycans. A further extension of this technique has been the ability of ion mobility to separate isobaric fragment ions whose different gas-phase conformations has, for example, enabled Lewis epitopes and blood group antigens to be differentiated in positive ion mode [41]. This paper extends the above methods to an examination of the ability of ion mobility to extract structural information from fragments of high-mannose and other *N*-glycans generated from negative molecular ions produced in the trap region of a Waters traveling wave ion mobility Synapt G2Si mass spectrometer [52].

Most of the work so far reported on ion mobility of negative ion collision-induced dissociation (CID) fragmentation of *N*-glycans has been performed in the transfer region of the instrument and has been reported in detail in previous publications [48–51] . Briefly, major ions arise from ^2,4^A cross-ring cleavages of the GlcNAc residues of the chitobiose core and from a B cleavage between these residues. These ions define the presence or absence of core fucose residues. Branching patterns are defined by several specific ions. An ion, termed ion D, formed by loss of the 3-antenna and chitobiose core, accompanied by a further ion formed by loss of water (termed the D-18 ion) and two cross-ring cleavage fragments of the branching mannose (^0,3^A and ^0,4^A fragments) define the composition of the 6-antenna. A pair of ions at *m/z* 485 and 467 are present in the spectra of high-mannose glycans when a mannose residue (mannose 8) is present on the 6-branch of the 6-antenna, as in the spectra of compounds **5**, **6**, **8**-**12, 14**, and **15**. Triantennary isomers can be differentiated by the masses of these ions and by the presence or absence of an ion at *m/z* 831 that is diagnostic for the structure **23** [53], and bisected glycans (possessing a GlcNAc residue attached to the 4-position of the branching mannose as in Compound **24**) can be identified by the presence of a prominent D-221 fragment. Antenna structure is revealed by cross-ring cleavage ions such as Gal-GlcNAc-O-CH=CH-O^-^ (*m/z* 424) and, finally, C_1_-Type fragments identify the residues terminating the antennae. Trap fragmentation spectra contain these ions but secondary fragments resulting from glycosidic cleavages of the major ^2,4^A fragments from the core GlcNAc residues are more prominent. Most of the glycan fragment peaks are composed of several structures and, as we [41] and others [54, 55] (and see the review by Hofmann and Pagel [7]) have shown for positive ion spectra, these structures can often be separated by ion mobility to provide structural information additional to that contained within the CID spectra themselves. Fragment ion structures generated from the high-mannose glycans in negative ion mode have not been studied before and are the topic of this paper. Reference to the hybrid and complex glycans is made when additional structural information can be obtained from their fragment ions.

## Materials and Methods

Synthetic high-mannose glycans (Compounds **1**-**4**, **6**, **9**, **12**) were purchased from Dextra Laboratories, (Reading, UK). Compounds **2**-**6** and **9** were also released from ribonuclease B [56, 57] by hydrazinolysis and re-acetylated [58, 59], and additionally released from gp120 from human immunodeficiency virus with peptide *N*-glycosidase F (PNGase F) as described earlier [60, 61]. Compound **3** was also obtained from chicken ovalbumin [62–64]. The d1d1d2 isomer of Man_8_GlcNAc_2_ (**7**) was a gift from Drs. Isabelle Chantret and Stuart Moore, Paris, France) and the d1d2d3 isomer (**8**) was from Dr. R. Raquel Montesino Sequí (Concepción, Chile). High-mannose glycans **10** and **11** were from *Saccharomyces cerevisiae* [65] and their structures were confirmed by negative ion CID. Hybrid glycans (**16**-**20**) were from gp120 produced recombinantly in the presence of the α-mannosidase inhibitor swainsonine [66], and compounds **13**-**15** were from gp120 produced recombinantly in the presence of the α-glucosidase inhibitor *N*-butyldeoxynojirimycin (NBDNJ) [67, 68]. The bisected glycan (**24**) was released from chicken ovalbumin by hydrazinolysis; the complex glycans (**21** and **22**) were released from porcine thyroglobulin [69, 70] and bovine fetuin [71] by hydrazinolysis and from gp120 as described above. The biantennary glycan Gal_2_Man_3_GlcNAc_4_Fuc_1_ (**21**) was also obtained from Oxford GlycoSystems (Abingdon UK).

### Mass Spectrometry

Synthetic glycans (1μg/μL) and hydrazine-released glycans (similar but unmeasured concentration) were used without further purification; the PNGase F-released glycans (from about 20 μ of glycoprotein), in 2 μL of water were cleaned with a Nafion membrane [72] prior to analysis. All glycans were dissolved in 1:1 (v:v) water:methanol (about 6 μL) to which had been added about 0.2 mL of an 0.5 mM solution of ammonium phosphate (to form phosphate adducts of the glycans). Samples were infused into the nano-electrospray (nano-ESI) source of a Waters Synapt G2Si traveling wave ion mobility (TWIMS) Q-TOF mass spectrometer (Waters, Manchester, UK) through gold-coated borosilicate capillaries prepared in-house [73]. The instrument was configured as follows: capillary voltage, 0.8–1.0 kV; sample cone, 150 V; extraction cone, 25 V; cone gas, 40 L/h; source temperature, 80 °C; trap collision voltage, 50–160 V (dependent on the mass of the target ion); transfer collision voltage, 4 V; trap DC bias, 60 V; ion mobility wave velocity, 450 m/s; ion mobility wave height, 40 V; trap gas flow, 1.6 mL/min; ion mobility gas flow, 80 mL/min. Spectra were acquired for about 2 min for most samples although longer time was required with some of the biological samples (normally 2–5 min although compound 8 required 22 min). Data was acquired and processed with MassLynx ver. 4.1 and Driftscope ver. 2.8 software (Waters). The nomenclature used to describe the fragment ions is that devised by Domon and Costello [74] with the addition of ion D (described above) and the use of the subscript R (for reducing terminus) to describe fragments of the reducing-terminal GlcNAc residue and R-1 to describe fragments from the other core GlcNAc. This nomenclature simplifies description of the fragmentation processes because it avoids the subscript number changing with different antenna chain lengths. Estimated rotationally averaged collision cross-sections (CCSc) were calculated by fitting the arrival time distributions (ATDs) to a single or double Gaussian distribution. A dextran calibration ladder with known absolute CCSs that were measured on a drift tube instrument was used for estimating *N*-glycan traveling wave CCS values as previously described [75, 76].

## Results and Discussion

In order to generate the CID spectra, the *N*-glycans are normally adducted with suitable anions to render them sufficiently stable to be transmitted to the collision cell and thus avoid extensive fragmentation in the ion source as is common with [M-H]^-^ ions [49]. Phosphate was chosen in this case because this anion is normally present in material derived from biological sources and provides a convenient stabilizing anion. Chloride is also a common constituent and a satisfactory anion but sensitivity can be compromised by the presence of the chlorine isotopes. Both anions, however, yield identical CID spectra because the first stage of fragmentation involves abstraction of a proton by the anion to leave a selection of [M-H]^-^ ions whose further fragmentation appears to be independent of the anion. Figure 1 shows the trap fragmentation of a typical high-mannose glycan (Man_9_GlcNAc_2_ **9**). The inset shows the nomenclature used to describe various parts of the molecule. Most of the ions discussed in this paper arise as fragments of the ^2,4^A_R_ and ^2,4^A_R-1_ ions by losses of mannose residues, normally involving additional cross-ring fragmentation. These ions are identified in Figure 1 by red and blue arrows, respectively. Also in Figure 1 are two extracted ATDs of the fragments at *m/z* 647 and 707, which show separated structures. Ions are discussed below in order of the number of constituent hexose residues.

### Hexose_2_

#### Fragments of Composition Hex_2_GlcNAc_1_-O-CH=CH-O^-^ (m/z 586)

**Figure 2:**
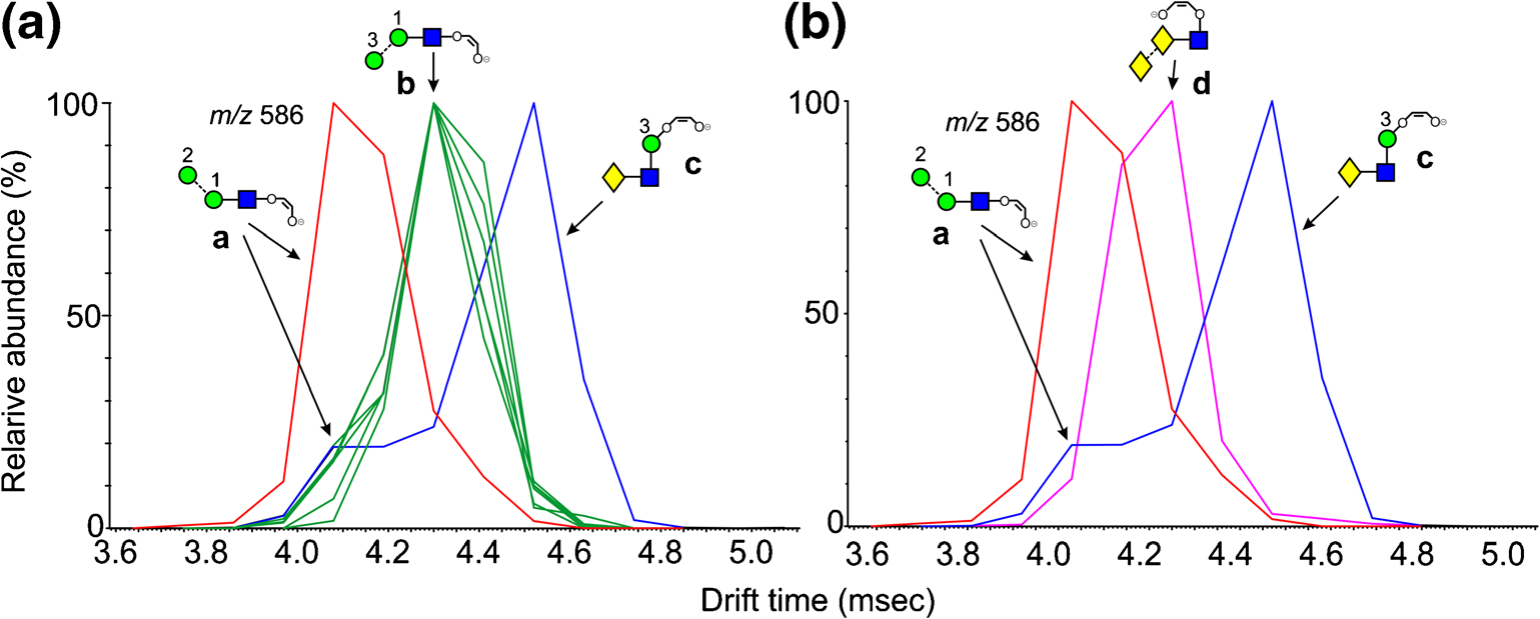
Extracted fragment ATDs of Hex_2_-containing ions. (**a**) Fragments of composition Hex_2_GlcNAc_1_-O-CH=CH-O^-^ (*m/z* 586). Green traces show the extracted fragment ATDs of ion **b** found in most of the glycans. The red trace is the ATD from Man_3_GlcNAc_2_ (ion **a**) and the blue trace is that from glycans containing a Gal-GlcNAc antenna [in this case from the biantennary glycan **21** (ion **c**)]. (**b**) The same traces from ions **a** and **c** together with ion **d** which is produced from glycans carrying an α-galactose residue terminating an antenna (pink trace, from glycan **22**)

The ion at *m/z* 586 was a minor ion in the spectra of most glycans except Man_3_GlcNAc_2_ (**1**) and the α-galactosylated biantennary glycan **22**. Nevertheless it displayed several drift times from the various glycans indicating different structures. Extracted fragment ATDs of this ion from various *N*-glycans are shown in Figure 2. Estimated cross-sections are listed in Table 1. There is the possibility that the ATDs shown in the figures represent the average of unresolved conformers. However, in most cases the peak widths suggest otherwise. If conformers do exist, they would only be able to be resolved with instruments with much higher IMS resolving power. Thus, the CCS values reported here are appropriate to most existing ion mobility instruments. Measurements made on the same ion from different compounds rarely differed by more than 1 Å^2^. Two structures (**a** and **b**, Figure 2a) are possible for this ion derived from the high-mannose glycans **1**-**15**. The extracted fragment ATD of *m/z* 586 from glycans **2**-**15** gave a single peak (green trace). A single cleavage, namely loss of the 6-antenna (mannoses 2, 6 and 7), would yield ion **b** from Man_5_GlcNAc_2_ (**2**)_._ Two cleavages, namely loss of the 6-antenna together with d1 mannose residues 3, 4, and 5 (see Figure 1) would produce this ion from the larger glycans. Man_3_GlcNAc_2_ (**1**) where this ion was prominent, on the other hand, produced a second ion at a shorter drift time which, therefore, must have structure **a**. Complex, biantennary glycans with Gal-GlcNAc-antenna (e.g., **21** and **22**) and the hybrid glycan (**20**) produced a third ion at a longer drift time. This latter ion presumably arose from the antennae and has the structure **c** (Figure 2a). In the spectra of the fucosylated biantennary glycans from parotid glycoproteins, the ion with structure **c** was absent when both antennae contained fucose, confirming the structure and demonstrating the stability of the fucose residues in the negative ion spectra. In positive ion mode, major fragments arose from these antennae-fucosylated glycans by successive losses of fucose.

**Table 1:**
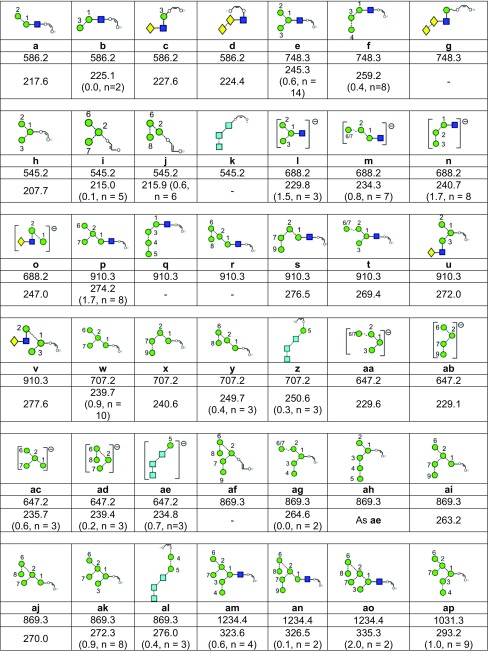

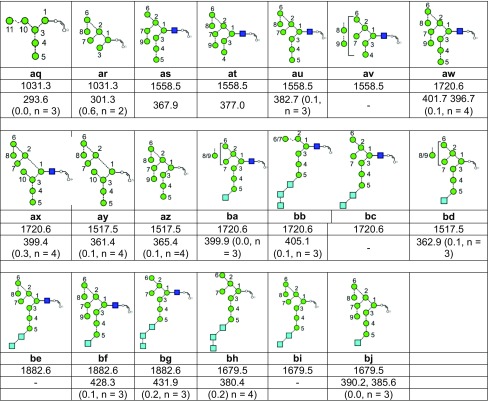
Structures, Masses, and Estimated nitrogen Traveling Wave Collisional Cross-Sections of the Fragment Ions^a^

^a^For each ion the table shows the glycan structure, the ion number (in bold), the mass and the collisional cross-section in Å2. Where multiple measurements of the CCS values were obtained, the error (from the STDEV function of Microsoft Excel) and the number (n) of measurements is given.

The negative ion CID spectra of complex glycans containing an α-galactose residue terminating the antennae (e.g., **22** from porcine thyroglobulin) produce a very prominent ion at *m/z* 586 (pink trace) with the structure **d** (Figure 2b) and a drift time intermediate between ions **a** and **c**. The presence of α-galactose residues on the termini of the antennae of complex glycans is of interest to investigators developing therapeutics because of its potential antigenic properties and, consequently, the collision cross-section of *m/z* 586 provides an alternative way of confirming the presence of this feature.

Similar behavior to the above was seen for the corresponding ion lacking the GlcNAc residue (*m/z* 383) but the separation between the peaks was smaller than that for *m/z* 586 and is not shown.

### Hexose_3_

#### Fragments of Composition Hex_3_GlcNAc_1_-O-CH=CH-O^-^ (m/z 748)

**Figure 3:**
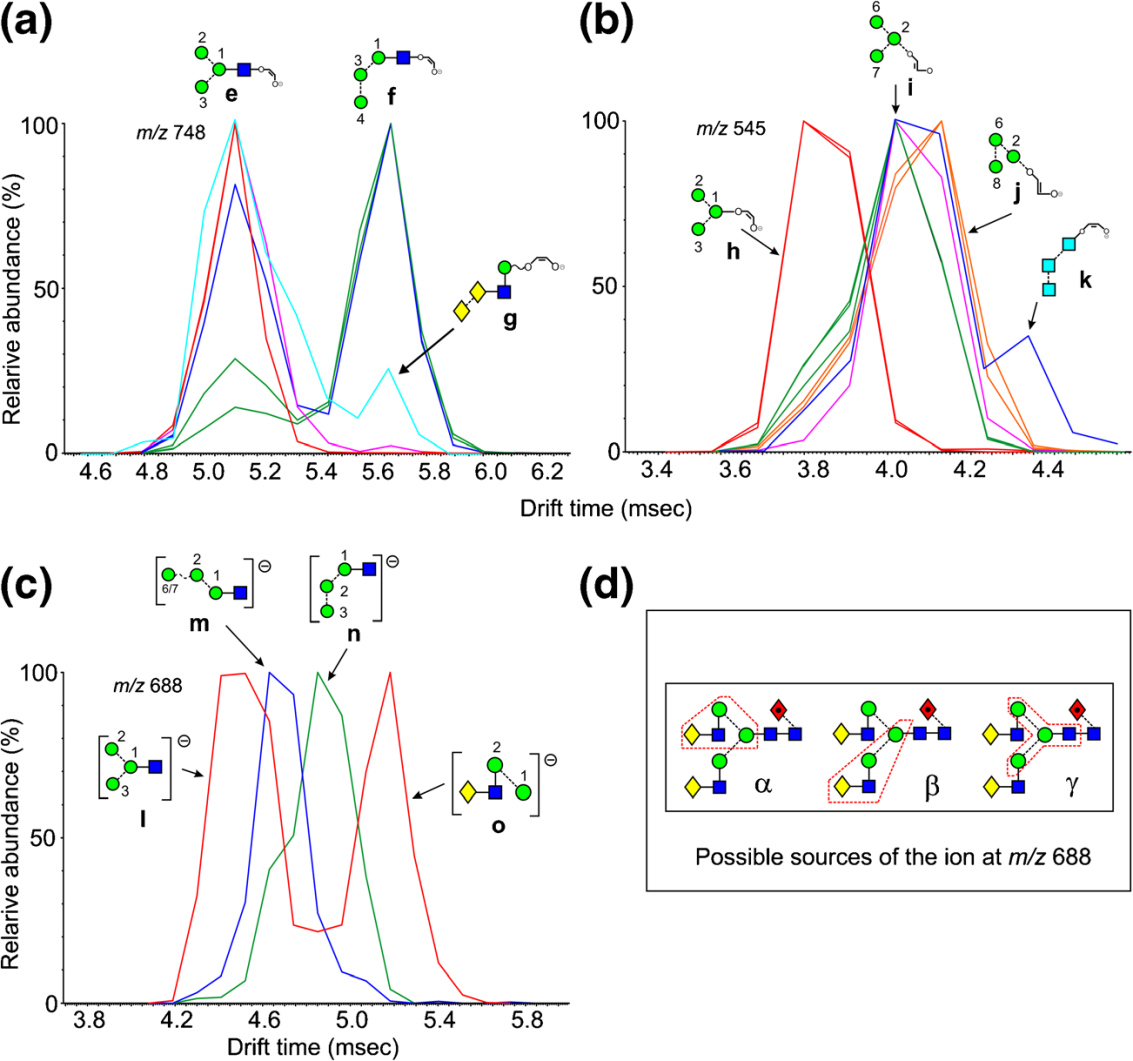
Extracted fragment ATDs of Hex_3_-containing ions. **(a)** Fragments of composition Hex_3_GlcNAc_1_-O-CH=CH-O^-^ (*m/z* 748). Red traces (ion **e**), extracted fragment ATDs from Man_3_GlcNAc_2_ (**1**), Pink trace, Ion **e** from Man_5_GlcNAc_2_ (**2**) and the complex and hybrid glycans. Blue trace, extracted fragment ATD from Man_6_GlcNAc_2_ (**3**), (ion **f**). Light blue trace, ion **g** from Gal_2_GlcNAc_4_Fuc_1_ (**21**). **(b)** Fragments of composition Hex_3_-O-CH=CH-O^-^ (*m/z* 545). Green trace, ATD of ion **h** produced by Man_5-7_GlcNAc_2_ (**2**, **3**, **4**). Orange trace, ion **I** from glycans with at least mannose 4 in the 3-antenna. Pink trace, ATD from Glc_1_Man_9_GlcNAc_3_ (**12**). Red trace, ion **g** produced by Man_3_GlcNAc_2_ (**1**) and the complex glycans. Blue trace, second peak, ion **k** containing the three glucose residues (from Compound **14**). **(c)** Ion **l** (red trace, first peak) arises from cleavage γ in panel 3d from the biantennary glycan and from Man_3_GlcNAc_2_ (**1**). The second peak of the red trace is ion **o** produced by cleavage α (panel 3d), also known as the diagnostic D ion. Ions **m** and **n** are produced by many of the hybrid and high-mannose glycans [e.g., blue trace from Man_5_GlcNAc_2_ (**2**), green trace (from Man_9_GlcNAc_2_ (**9**)] from glycans with mannose 4 in the 3-antenna). **(d)** Structure of the biantennary glycan Gal_2_Man_3_GlcNAc_4_Fuc_1_ (**21**) showing cleavages α, β, and γ leading to the fragment ion at *m/z* 688 enclosed in the red boxes

Extracted fragment ATDs of *m/z* 748 from various *N*-glycans are shown in Figure 3 and estimated CCSs are listed in Table 1.The extracted fragment ATD from this ion derived from Man_3_GlcNAc_2_ (**1**) where it was the ^2,4^A_R_ ion, gave a single peak with structure **e** (Figure 3a, red trace, CCS = 245.3 Å^2^ ± 0.6, n = 14). Man_5_GlcNAc_2_ (**2**, pink trace) also produced this ion by presumably releasing mannoses 6 and 7. The hybrid glycans also produced a single ion at the same drift time confirming the absence of additional mannose residues in the 6-antenna. Man_6_GlcNAc_2_ (**3**) produced two equally abundant well separated peaks (blue trace), the second of which could be formed by a single cleavage (losses of mannoses 2, 6, and 7) to give ion **f**. This ion is essentially linear, whereas the other is branched, probably explaining its longer drift time. Ions with these two ATDs were produced in varying relative abundance from all of the other high-mannose glycans [green traces from Man_8_GlcNAc_2_ (**6**) Man_9_GlcNAc_2_ (**9**) and Glc_1_Man_9_GlcNAc_2_ (**12**)]. Thus the presence of the well-separated second peak appears to be diagnostic for mannose substitution in the 3-antenna and is useful because no fragment has been found in the CID spectrum that is diagnostic of this substitution. Hybrid glycans produced only ion **e**. Two equally abundant peaks with the same two drift times as those from the above glycans were produced from the α-Gal-containing glycan (**22**). Clearly the second peak could not have the structure **f** and, thus, it was assigned the structure **g**. The reference sample of the biantennary glycan **21** gave a single peak corresponding to ion **e**. However, the same compound from thyroglobulin produced a second peak (light blue trace) with the drift time of ion **g**. This sample is the source of the α-galactose substituted biantennary glycan **22** and, although the presence of an isomer (structure **23**) of the biantennary glycan **21** has been suspected to occur in this sample, no conclusive evidence for its existence has been found. The presence of the mobility peak at the drift time of this ion, therefore, strongly supports its existence. Undoubtedly, this ion and ion **f** have slightly different drift times but the resolution of current instrumentation is insufficient to resolve them.

#### Fragments of Composition Man_3_-O-CH=CH-O (m/z 545)

The corresponding ion lacking the GlcNAc residue (*m/z* 545), however, showed a slightly different behavior. It produced essentially a single peak (green trace, Figure 3b) from the high-mannose glycans Man_5-7_GlcNAc_2_ (**2**, **3**, **4**, ion **i**) but a second one with a slight shift to higher drift times from the d1d1d3 isomer of Man_8_GlcNAc_2_ (**6**) and Man_9_GlcNAc_2_ (**9**, orange trace). This latter peak would, therefore, appear to have structure **j**. Glc_1_Man_9_GlcNAc_3_ (**12**, pink trace) gave a broader peak consistent with the presence of ions **i** and **j**. Man_3_GlcNAc_2_ (**1**) and the hybrid glycan (**16**) produced an earlier peak (red trace, **h**), coincident with the shoulder on the main peak (**i**) from the other high-mannose glycans. The structure (**h**) is confirmed by its presence as the ^2,4^A_R-1_ ion from Man_3_GlcNAc_2_ (1). The three Glc_3_-containing glycans (**13**, **14,** and **15**) produced a minor fourth peak at a longer drift time (blue trace from compound **14**) attributed to the linear structure **k**.

### Fragments of Composition Hex_3_GlcNAc_1_ (m/z 688)

An important feature of the negative ion CID spectra of *N*-glycans is the presence of a series of ions specific to the 6-antenna and, thus, revealing its composition. Elimination of the chitobiose core and the 3-antenna yields an ion containing the 6-antenna together with the core branching mannose residue (mannose 1, Structure α in Figure 3, panel d). This ion has been named the D ion and is accompanied by another ion formed by additional loss of water (the D-18 ion) and two cross-ring cleavage ions (^0,3^A and ^0,4^A) of the branching mannose as described in the Introduction. The D ion from biantennary glycans such as **21** and **22** has the composition Gal_1_Man_2_GlcNAc_1_ (Hex_3_GlcNAc_1_) but ions of this composition can arise from other parts of the molecule as shown in Figure 3d, structures β and γ. Ions at *m/z* 688 are, thus, often seen in the spectra of *N*-glycans even though they do not have the structure of the D ion. Consequently, it would be useful to have a means to confirm if an ion at this mass was indeed a D fragment. Partial confirmation is provided by the presence of accompanying D-18 and sometimes D-36 ions but this correlation is not considered absolute. The extracted fragment ATD of *m/z* 688 from the trap fragmentation spectrum of the biantennary glycans (**21** and **22**) demonstrated two well-defined peaks (Figure 3c, red trace). The broad peak with the shortest drift time (collision cross-section 231.3 Å^2^) was similar to that (229.8 Å^2^) from Man_3_GlcNAc_2_ (**1**) and, therefore, has the structure **l**. Hybrid and high-mannose glycans, on the other hand, produced one of two peaks with drift times intermediate between these two peaks. Man_5_GlcNAc_2_ (**2**) produced a peak with a cross-section of 234.4 Å^2^ to which structure **m** was assigned (blue trace), whereas the larger compounds with mannoses 4 and 5 in the 3-antenna produced, in addition, a peak with a slightly longer drift time which was probably **n** (green trace). The absence of ions corresponding to structure **o** (red trace) in the spectra of the hybrid glycans **16**, **18,** and **20** that contained the Gal-GlcNAc-Man- group clearly showed that the Gal-GlcNAc-Man-Man ion (*m/z* 688) in biantennary glycans arose mainly from the 6- and not the 3-antenna. Thus, the peak with the longest drift time from the biantennary glycans appeared to have structure **o** and to be diagnostic for the presence of the D ion. Furthermore, the major fragments of *m/z* 688, obtained by acquisition of transfer fragmentation spectra of the mobility extracted ion at *m/z* 688, were *m/z* 670 and 652, corresponding to D-18 and D-36, respectively. The D-18 ion also produced a doublet but the ion at *m/z* 652 only produced a singlet.

### Hexose_4_

#### Fragments of Composition Hex_4_GlcNAc_1_-O-CH=CH-O^-^ (m/z 910)

**Figure 4:**
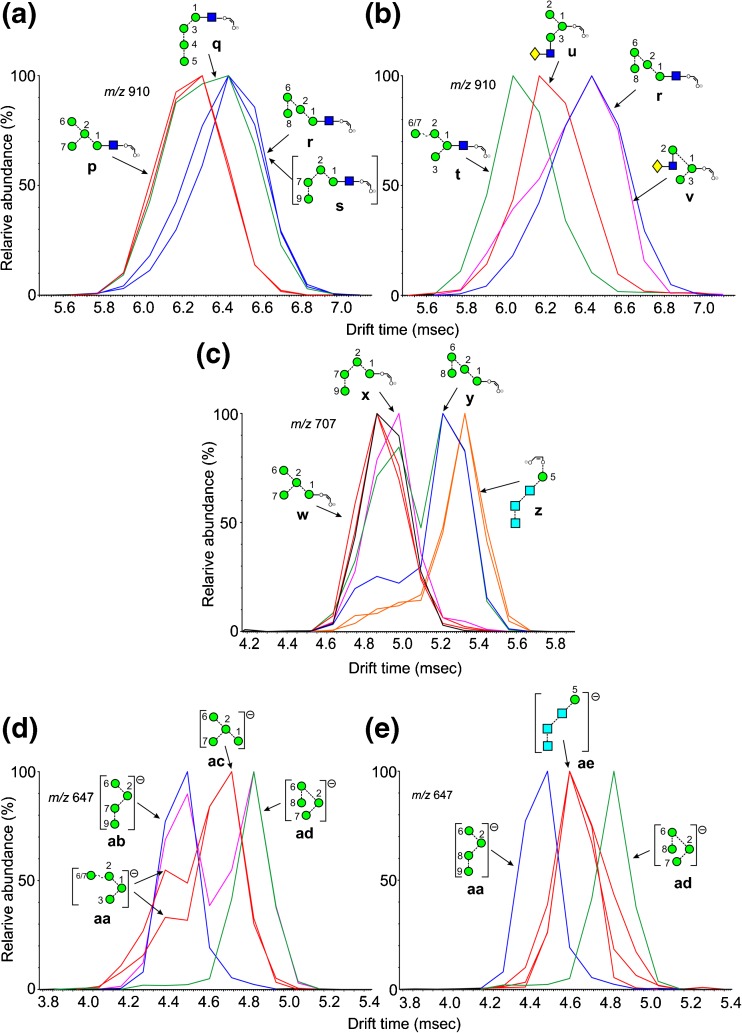
Extracted fragment ATDs of Hex_4_-containing ions (*m/z* 910). **(a)** Fragments of composition Hex_4_GlcNAc_1_-O-CH=CH-O^-^ (*m/z* 910). Red trace, ion **p** from Man_5,6_GlcNAc_2_ (**4**, **5**). Green trace, combination of ions **p** and **q** from the d1d1-isomer of Man_7_GlcNAc_2_ (**7**). Blue traces, ions **r**, **s,** and possibly **q** from the larger glycans Man_8_GlcNAc_2_ (**6**-**8**) and Man_9_GlcNAc_2_ (**9**). **(b)** Red trace ion **u** from hybrid (glycan **20**) and complex glycans. Green trace, ion **t** from the hybrid glycan Man_4_GlcNAc_3_Fuc_1_ (**15**). Pink trace, ions **u** and **v** from the biantennary glycan (**21**). The blue trace is from Man_9_GlcNAc_2_ (**9**) and is the same as in panel a. **(c)** Fragments of composition Hex_4_-O-CH=CH-O^-^ (*m/z* 707). Red traces, ion **w** from glycans Man_5_GlcNAc_2_ (**2**), Man_6_GlcNAc_2_ (**3**), and several other glycans and formed by elimination of the 3-antenna. Pink trace, ion **x** from glycans with at least mannose 4 in the 3-antenna. Green trace (doublet), ion **x** and ion **y** from Man_9_GlcNAc_2_ (**9**). Blue trace, ion **y** from Man_8_GlcNAc_2_ (**6**) Orange trace, ion **z** from glycans **13**, **14**, and **15**, containing three glucose residues in the 3-antenna. **(d** and **e)** Extracted fragment ATDs from *m/z* 647. **(d)** Red traces, ions **aa** and **ac** from Man_5_GlcNAc_2_ (**2**) and Man_6_GlcNAc_2_ (**3**). Ion **ac** is also known as ion D. Green trace, ion **ad** from glycans with mannose 8 in the 6-antenna (from Man_7_GlcNAc_2_ (**5**). Blue trace, ion **ab** from the d1d1d2 isomer of Man_8_GlcNAc_2_ (**7**). Pink trace, doublet from Man_9_GlcNAc_2_ (**9**). (**e**). Blue and green traces as in panel a together with traces from ion **ae** from glycans containing Glc_3_ groups

Extracted fragment ATDs of *m/z* 910 from various *N*-glycans are shown in Figure 4. The ATD profile of *m/z* 910 from Man_5-7_GlcNAc_2_ (**2**-**4**) and the hybrid glycans **19** and **20**, i.e., those glycans lacking the α1→2 mannose residues 8 and 9 gave a single peak (ion **p**, red traces, Figure 4a), whereas that from the various isomers of Man_8_GlcNAc_2_ (**6**-**8**) and Man_9_GlcNAc_2_ (**9**) with mannose residues 8 and 9 produced a peak with a slightly greater drift time (blue traces, panel a). This behavior is consistent with the occurrence of structures **r** and/or **s**. Assignment of structure **r** is consistent with the general observation that the presence of mannose 8 leads to longer drift times and evidence for ion **s** is provided by the presence of an ion at this drift time from the d1d1d2-isomer of Man_8_GlcNAc_2_ (**7**). The broad, green trace from the d1d1-isomer of Man_7_GlcNAc_2_ (**5**) appeared to contain ions **p** and **q**, both of which could be formed from a single cleavage from the ^2,4^A_R_ fragment. Support for the structure of ion **q** was the observation that it was seen in the spectra of glycans having mannoses 4 and 5 in the 3-antenna but which lacked the α1→2 mannoses 8 and 9. An ion with the drift time of ion **s** was also prominent in the spectrum of the d1d1d2 isomer of Man_8_GlcNAc_2_ (**7**). The broadness of the peaks (blue traces) was consistent with some or all of these structures being present. The hybrid glycan, Man_6_GlcNAc_3_Fuc_1_ (**20**), produced a third peak (red trace, Figure 4b) with a slightly shorter drift time than that produced by Man_5_GlcNAc_2_ (**2**) even though it contained the same 6-antenna. This peak was coincident with the early part of a broad peak from the biantennary glycan Gal_2_Man_3_GlcNAc_4_Fuc_1_ (**21**, pink trace, Figure 4b), which appeared to consist of the ions **u** and **v**. Clearly, this latter compound does not have four attached hexose residues leaving the structures **u** and **v** as the only possible ions. The hybrid glycan Man_4_GlcNAc_3_Fuc_1_ (**17**) produced yet another peak (green trace) with an even shorter drift time. Because this ion contains all four mannose residues from this glycan it must have structure **t**.

#### Fragments of Composition Hex_4_-O-CH=CH-O^-^ (m/z 707)

The corresponding ion lacking the GlcNAc residue (*m/z* 707) also displayed several structures. A single extracted fragment ATD was observed from Man_5_GlcNAc_2_ (**2**), Man_6_GlcNAc_2_ (**3**, red traces, Figure 4c), the d1d1 isomer of Man_7_GlcNAc_2_ (**4**), and the Man5-containing hybrid glycans (**19** and **20**), consistent with structure **w** formed by elimination of the 3-antenna from the ^2,4^A_R-1_ ion. The d1d3 isomer of Man_7_GlcNAc_2_, (**5**) the d1d1d3 isomer of Man_8_GlcNAc_2_ (**6**, blue trace), Man_9_GlcNAc_2_ (**9**, green trace, illustrated in Figure 1), and Glc_1_Man_9_GlcNAc_2_ (**12**, not shown) produced a well-separated second peak consistent with structure **y**. The longer drift time produced by the presence of mannose residue 8 in the 6-antenna was consistent with earlier observations made above and on the parent compounds [60]. Man_9_GlcNAc_2_ (**9**), the d1d1d2 isomer of Man_8_GlcNAc_2_ (**7**, pink trace), and Glc_1_Man_9_GlcNAc_2_ (**12**, not shown) produced a third peak that was just separated from the first to which structure **x** was assigned. This ion could be produced from the ^2,4^A_R-1_ fragment by cleavage of the 3-antenna (mannoses 3, 4, and 5) together with loss of mannoses 6 and 8. Although the separation between the first (red) and second (pink) peaks was only marginal, it was observed consistently on repeat experiments. All three of the glycans containing three glucose residues in the 3-antenna (**13**-**15**) produced an additional ion at longer drift times. This ion appears to have the structure **z** and has been observed as a major diagnostic ion in the transfer fragmentation spectra of these compounds.

#### Fragments of Composition Man_4_ (m/z 647)

This was the only ion of the Hex_n_ series that showed any significant drift time differences among the various possible isomers and is an important ion because it is a potential D ion (structure **ac**, Figure 4d) from hybrid and complex glycans containing mannoses 2, 6, and 7 in the 6-antenna. However, as with the D ion from the biantennary glycans (*m/z* 688), discussed above, *m/z* 647 can also be produced from other regions of the molecules and its positive identification as a D ion by ion mobility would be useful. A major and a minor peak were recorded from this ion from Man_5_GlcNAc_2_ (**2**), Man_6_GlcNAc_2_ (**3**, Figure 4d, red traces), and from the d1d1 isomer of Man_7_GlcNAc_2_ (**4**), which would be expected to produce a D ion at *m/z* 647. The ion from Man_7_GlcNAc_2_ (d1d3 isomer) (**5** green trace) and Man_8_GlcNAc_2_ (**6**), which have additional mannose residues in the 6-branch of the 6-antenna and for which *m/z* 647 is not the D ion, produced peaks with a longer drift time (green trace) consistent with the above observations. These peaks would appear to have structure **ad** as the result of a single cleavage from the ^2,4^A_R_ or ^2,4^A_R-1_ ions. A single peak with a shorter drift time than the other two, and consistent with structure **ab**, was recorded from the d1d1d2 isomer of Man_8_GlcNAc_2_ (**7**, blue trace). This ion was presumably produced by a similar single cleavage. The ion from Man_9_GlcNAc_2_ (**9**, pink trace and illustrated peak in Figure 1) also produced a peak with a similar drift time, together with a second at a drift time equivalent to that from the other glycans containing the 8-mannose in the 6-antenna (ion **ad**). The minor peaks with shorter drift times on the side of the trace (red) from ion D (structure **ac**) appear to have structure **aa**. All the three glucose-containing glycans (**13**-**15**) produced the same single ATD peak with a drift time (red traces, Figure 4e) slightly shorter than that produced by ion D. The equality of the drift time of *m/z* 647 from these three glycans suggests that all three ions had the same structure with ion **ae** being the obvious candidate.

The ATD peak from the D-18 ion (*m/z* 629) from Man_5_GlcNAc_2_ (**2**) and Man_6_GlcNAc_2_ (**3**) and from the corresponding ion in the spectra of the other high-mannose glycans was broad and ill-defined, suggesting water loss from several sites.

### Hexose_5_

#### Fragments of Composition Hex_5_-O-CH=CH-O^-^ (m/z 869)

**Figure 5:**
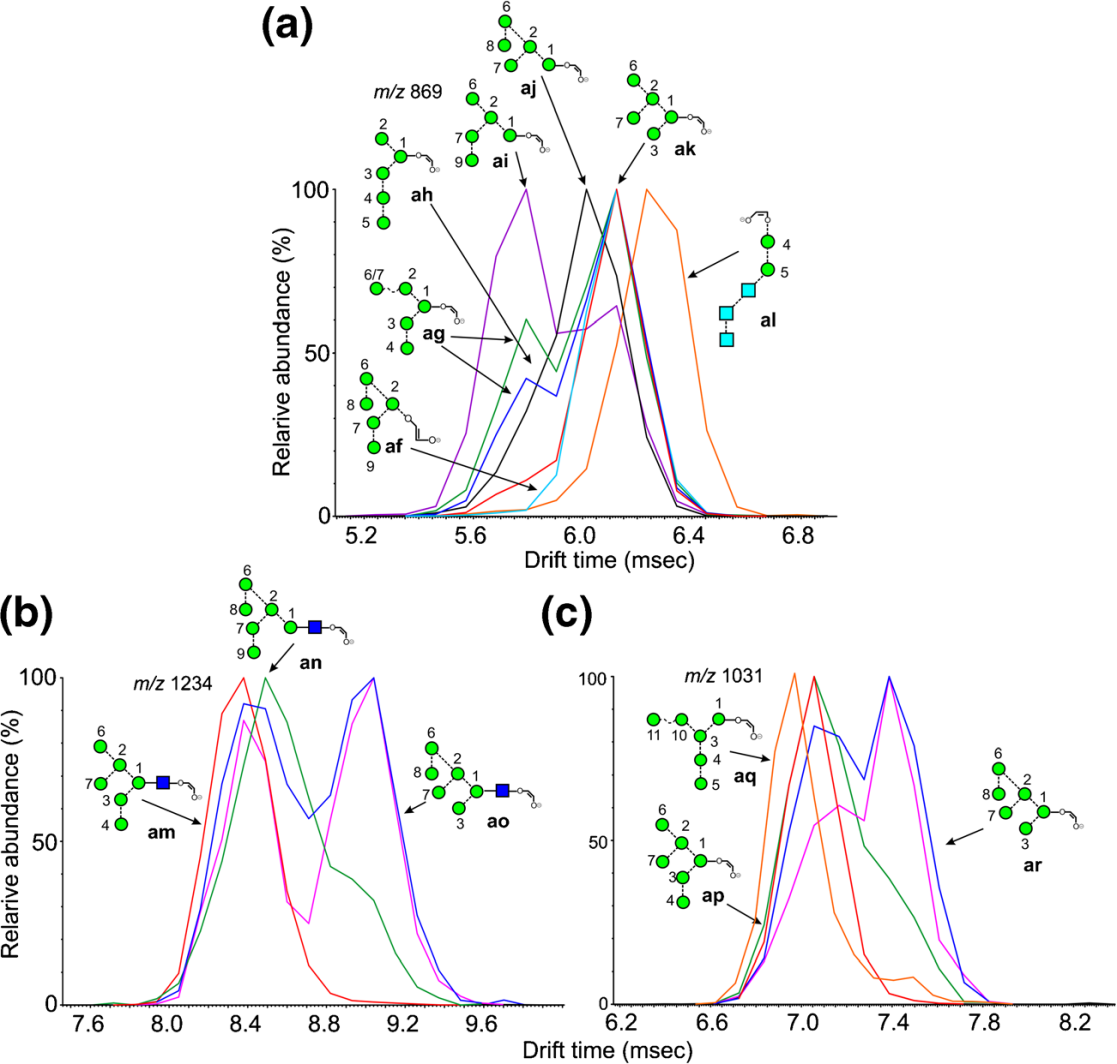
Extracted fragment ATDs of Hex_5_ and Hex_6_-containing ions. **(a)** Ions of composition Hex_5_-O-CH=CH-O^-^ (*m/z* 869). Red trace, ion **ak** from Man_5_GlcNAc_2_ (**2**). Blue and green traces, ions **ag** and **ah** from Man_6_GlcNAc_2_ (**3**) and Man_7_GlcNAc_2_ (**4**). Light blue trace, ion **af** from Man_9_GlcNAc_2_ (**9**). Purple trace from the d1d1d2 isomer of Man_8_GlcNAc_2_ (**7)**, major peak, ion **ai**. Black trace, ion **aj** from glycans containing mannose 8 in the 6-antenna. Orange trace, ion **al** from glycans **13**, **14**, and **15**, containing three glucose residues in the 3-antenna. **(b)** Fragments of composition Hex_6_GlcNAc_1_-O-CH=CH-O^-^ (*m/z* 1234). Red trace: ion **am** from Man_6_GlcNAc_2_. Blue trace, second peak, ion **ao** from the d1d1d3 isomer of Man_8_GlcNAc_2_ (**6**). Pink trace, ATD from Glc_3_Man_8_GlcNAc_2_ (**14**) containing ions **am** and **ao**. Green trace, ion **an** from Man_9_GlcNAc_2_ (**9**) and its glucosylated analogues (**12** and **15**). **(c)** Fragments of composition Hex_6_-O-CH=CH-O^-^ (*m/z* 1031) with structures similar to those in Panel a. Orange trace, ion **aq** from *S. cerevisiae*

Although very small separations were seen for the Man_5_GlcNAc_1_-O-CH=CH_2_-O^-^ ion (*m/z* 1072) from the different glycans, no definitive conclusions were drawn regarding their structures. However, the corresponding ion lacking the GlcNAc residue (*m/z* 869, Figure 5a) produced separated isomers, Man_5_GlcNAc_2_ (**2**, red trace), Man_6_GlcNAc_2_ (**3**, blue trace), Man_7_GlcNAc_2_ (d1d1-isomer, **4**, green trace), Man_9_GlcNAc_2_ (**9**), Glc_1_Man_9_GlcNAc_2_ (**12**), and the hybrid glycan Gal_1_Man_5_GlcNAc_2_ (**20**) produced a prominent peak with a CCS of 272.3 Å^2^ ± 0.9 (n = 8). This ion is the ^2,4^A_R-1_ ion from Man_5_GlcNAc_2_ and, therefore, must have structure **ak**. Although this structure is consistent with the structures of glycans **3**, **4,** and **10**, the single symmetrical peak from Man_9_GlcNAc_2_ (**9**, light blue) is more likely to have structure **af** because it can be formed in a single cleavage from the molecular ion by an ^0,4^A_3_ cleavage of mannose 1. Support for this proposal comes from the spectrum of the mono-glucosylated Man_9_-analogue (**12**), which produced a single peak at the same drift time.

A minor peak appeared on the shorter drift time side of this peak in the spectrum of Man_6_GlcNAc_2_ (**3**) (blue trace) and a larger one was present in the spectrum of the d1d1-isomer of Man_7_GlcNAc_2_ (**4**, green trace). This peak was absent from the spectra of Man_9_GlcNAc_2_ (**9**) and the glucosylated analogue (**12**). Given that the difference in structure between Man_5_GlcNAc_2_ (**2**) and Man_6_GlcNAc_2_ (**6**) is the presence of the mannose at position 4, this indicates that the ion has the structure **ag**. An ion at the same drift time but with a higher relative abundance was present in the spectrum of the d1d1-isomer of Man_7_GlcNAc_2_ (**6**, green trace). This ion could have the same structure, produced by loss of two single mannose residues from the ^2,4^A_R-1_ ion or structure **ah**, also the product of two cleavages. The d1d1d2 isomer of Man_8_GlcNAc_2_ (**7**) also produced a major ion at this drift time (purple trace) but in this case the most likely structure is **ai** produced in a single cleavage from the ^2,4^A_R-1_ ion by loss of the 3-antenna (mannoses 3, 4, and 5). However, structures **ag** and **ah** cannot be discounted. Further ions with longer drift times were also present in the spectrum of this glycan but their structures are uncertain. The d1d1d3 isomer of Man_8_GlcNAc_2_ (**6**, black trace) and the d1d3 isomer of Man_7_GlcNAc_2_ (**5**) also produced broad peaks indicating the presence of several structures but with a maximum consistent with structures **aj,** which could also be formed in a single cleavage from the ^2,4^A_R-1_ ion.

An additional peak (orange trace) was observed from the three glycans with three glucose residues in the 3-antenna (**11**-**13**). As with *m/z* 707 discussed above, this ion is a major diagnostic ion for glycans with this composition and, because it is produced from all three of these glycans, it must have the structure **al**.

### Hexose_6_

#### Fragments of Composition Hex_6_GlcNAc_1_-O-CH=CH-O^-^ (m/z 1234)

Multiple structures were also produced from *m/z* 1234; extracted fragment ATDs are shown in Figure 5b. This ion is the ^2,4^A_6_ fragment from glycans of composition Man_6_GlcNAc_2_ (**3**) and is present as a single symmetrical peak from the d1d1 isomer (**3**, ion **am**, red trace). It is also present as a fragment in the spectra of various isomers of Man_7_GlcNAc_2_ (**4**, **5**) and Man_8_GlcNAc_2_ (**6**). In the spectrum of glycans carrying mannose 8 in the 6-antenna, i.e., the d1d3 isomer of Man_7_GlcNAc_2_ (**5**), the d1d1d3 isomer of Man_8_GlcNAc_2_ (**6**, blue trace), and Glc_3_Man_8_GlcNAc_2_ (**14**, pink trace), a second well-separated, abundant peak was present at higher drift times. The position of this peak is consistent with the incorporation of mannose 8 from the 6-antenna indicating structure **ao**. Peaks from the other compounds were broad but maxima from both Man_9_GlcNAc_2_ (**9**, green trace) and its glucosylated analogues (**12** and **15**) suggested structure **an** formed by the loss of the 3-antenna in a single cleavage from the ^2,4^A_R_ ion.

Similar profiles were observed from the ion lacking the GlcNAc moiety (*m/z* 1031) but with less separation between most of the peaks (Figure 5c). However, the ion at *m/z* 1031 from Man_10_GlcNAc_2_ (**11**) derived from *S. Cerevisiae* (orange trace) appears to have the structure **aq** formed by loss of the 6-antenna from the ^2,4^A_R-1_ ion.

### Hexose_7_

Only marginal separation was seen with various structures of ions containing seven mannose residues and no useful conclusions were drawn regarding their structures.

### Hexose_8_

#### Fragments of Composition Hex_8_GlcNAc_1_-O-CH=CH-O^-^ (m/z 1558)

**Figure 6:**
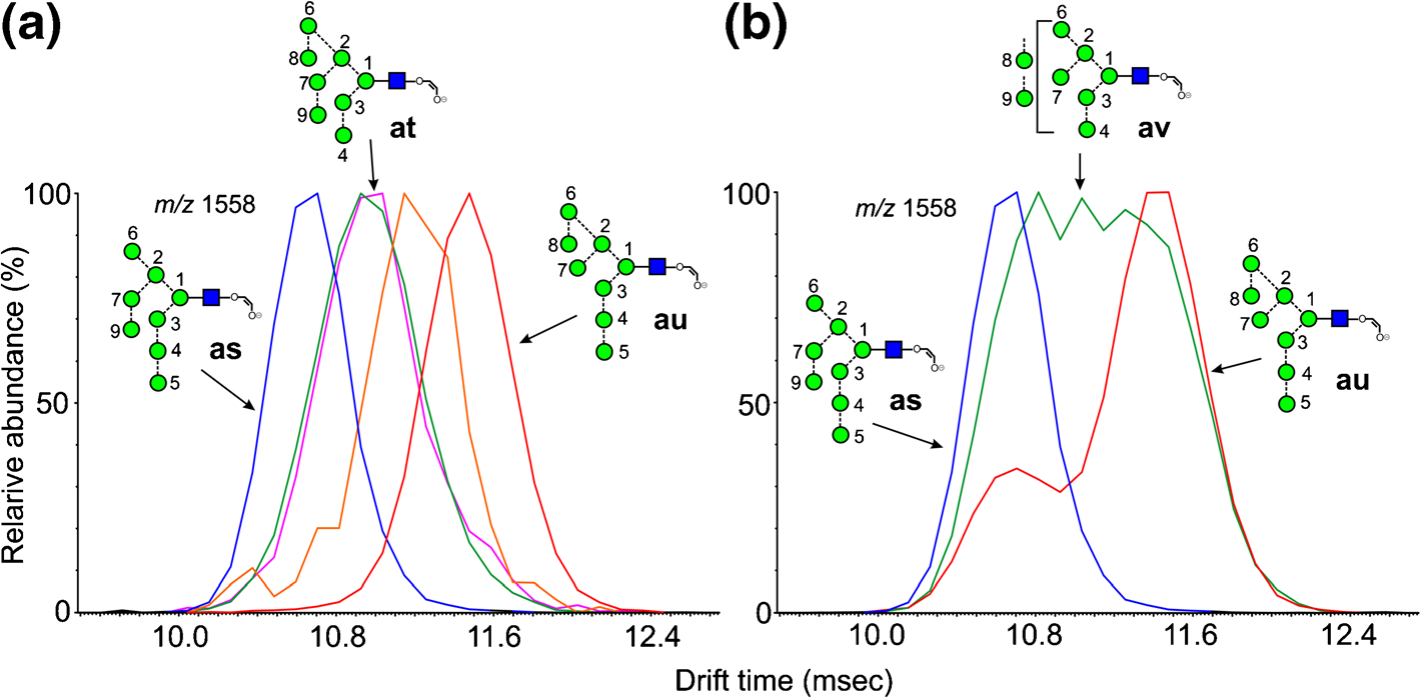
Extracted fragment ATDs of Hex_8_-containing ions (*m/z* 1558). **(a)** Fragments of composition Hex_8_GlcNAc_1_-O-CH=CH-O^-^ (*m/z* 1558). The red trace is the ^2,4^A_R_ ion (**au**) from the d1d1d3-isomer of Man_8_GlcNAc_2_ (**6**). The blue trace is the corresponding ion (**as**) from the d1d1d2-isomer of Man_8_GlcNAc_2_ (**6)** and the green trace (**at**) the ion from the d1d2d3 isomer. Glycans with extended 3-antennae (e.g., Glc_3_Man_9_GlcNAc_2_) also gave this ion (pink trace). The orange trace is a fragment of Glc_3_Man_7_GlcNAc_2_ but its structure is uncertain. **(b)** The blue trace (**as**) is the ^2,5^A_R_ ion from the d1d1d2-isomer of Man_8_GlcNAc_2_ (**6**) and the red trace is the corresponding profile of Man_8_GlcNAc_2_ from porcine thyroglobulin showing both isomers **6** and **7**. The green trace (**av**) is from Man_9_GlcNAc_2_ showing fragmentation by losses of each of the α-mannose residues

ATDs of fragments containing eight mannose residues are shown in Figure 6. The ion at *m/z* 1558 is the ^2,4^A_6_ ion from the Man_8_GlcNAc_2_ glycans (**6**-**8**). The d1d1d3 isomer of Man_8_GlcNAc_2_ (**6**) produced a single peak (Figure 6a, red trace), which has the structure of ion **au**. It was also produced from Glc_3_Man_8_GlcNAc_2_ (**14**) by loss of the three glucose residues. The corresponding ion **as** from the d1d1d2 isomer (**7**, blue trace) had a shorter drift time, consistent with the behavior shown by the parent compounds [60]. These two isomers can be distinguished by their characteristic negative ion CID spectra recorded in the transfer region of the Synapt instrument but the trap fragmentation spectra were virtually identical. In the transfer fragmentation spectrum, both the d1d1d2 (**7**) and d1d1d3 isomers (**6**) produce D, D-18, ^0,3^A_4_ and ^0,4^A_4_ ions at *m/z* 809, 791, 737, and 707, respectively. The glycans can be differentiated because the d1d1d3 isomer (**6**) produces a characteristic pair of ions at *m/z* 485 and 467, whereas the d1d2 isomer (**7**) does not. No diagnostic ions have been found to indicate the presence of the d1d1d2 isomer so that when *m/z* 485 and 467 are present, it is not always possible to say if the spectrum is from a mixture of both isomers, particularly as the relative abundance of *m/z* 485 and 467 varies with the collision energy. However, observation of the profiles of *m/z* 1558 in the trap fragmentation spectrum leaves no doubt as to which isomers are present. Figure 6b shows the profile from Man_8_GlcNAc_2_ obtained from a sample of glycans released from porcine thyroglobulin (red trace) showing the presence of both isomers.

Glc_1_Man_9_GlcNAc_2_ (**12**, green trace Figure 6a), Glc_3_Man_9_GlcNAc_2_ (**15**, pink trace), and the d1d2d3 isomer of Man_8_GlcNAc_2_ (**8**, green trace) produced a third ion whose structure must be **at**. This ion could also simply be formed from the glucose-containing glycans by a single elimination from the 3-antenna. Thus, the difference in drift time of the ^2,4^A_R_ from these three isomers of Man_8_GlcNAc_2_ (structures **as**, **at,** and **au**) allows them to be identified by their extracted fragment ATDs. A fourth ion was produced from Glc_3_Man_7_GlcNAc_2_ (**13**, orange trace). Several structures can be proposed but no evidence was found to determine which one(s) were present.

Ion mobility in the trap region also provided additional information on the fragmentation pathways shown by these ions. Thus *m/z* 1558 is formed from Man_9_GlcNAc_2_ (**9**) by loss of a mannose residue from one of the three antennae. The trap fragmentation profile of this ion as a fragment of Man_9_GlcNAc_2_ (**9**) is shown in Figure 6b (**av**, green trace) superimposed on the profiles from the d1d1d2 and d1d1d3 isomers of Man_8_GlcNAc_2_ (**7** and **6,** respectively). The broad, unresolved profile indicates the presence of several ions at this mass produced by loss of α-mannose residue from each antenna.

The fragments lacking the GlcNAc moiety (^2,4^A_R-1_ ion, *m/z* 1355) showed only marginal separation and were not considered analytically useful.

### Hexose_9_

#### Fragments of Composition Hex_9_GlcNAc_1_-O-CH=CH-O^-^ (m/z 1720)

**Figure 7:**
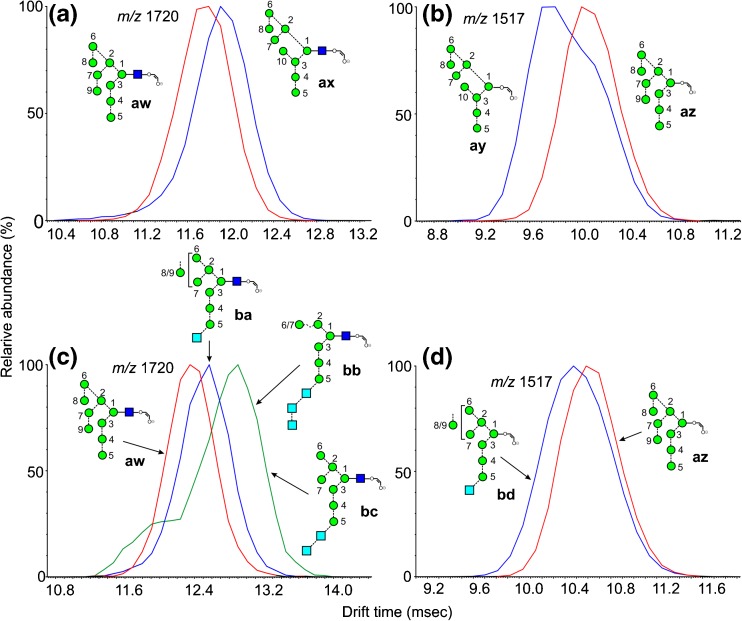
Extracted fragment ATDs of Hex_9_-containing ions. **(a)** Fragments of composition Hex_9_GlcNAc_1_-O-CH=CH-O^-^ (*m/z* 1720). Red trace, ^2,4^A_R_ ion (**aw**) from Man_9_GlcNAc_2_ (**9**). Blue trace, ion **ax** from the *S. cerevisiae*-derived glycan (**10**). **(b)** Corresponding ^2,4^A_R-1_ ions. **(c)** Red trace, ^2,4^A_R_ ion (**aw**) from Man_9_GlcNAc_2_ (**9**). Blue trace, ion **ba** as a fragment from Glc_1_Man_9_GlcNAc_2_ (**12**). Green trace, ions **bb** and **bc** as fragments from the Glc_3_-containing glycans. **(d)** Fragments of composition Hex_9_-O-CH=CH-O^-^ (*m/z* 1517). Red trace ^2,4^A_R-1_ ion (**az**) from Man_9_GlcNAc_2_ (**9**). Blue trace, ion **bd** as a fragment of Glc_1_Man_9_GlcNAc_2_ (**12**)

Two isomers of Man_9_GlcNAc_2_ (**9** and **10**) were available, giving *m/z* 1720 as the ^2,4^A_R_ ions with different cross-sections as shown in Figure 7a and b, and Table 1 (ions **aw** and **ax,** respectively). The extracted fragment ATD of ion **aw** from Man_9_GlcNAc_2_ (**9**) is shown as the red trace in Figure 7b. Loss of one of the mannoses from the 6-antenna was responsible for the formation of an isomeric ion with a slightly longer drift time than ion **aw** from Glc_1_Man_9_GlcNAc_2_ (**12**, ion **ba**, blue trace, Figure 7c) but the particular mannose that was eliminated was not determined. Loss of a hexose residue from Glc_3_Man_7_GlcNAc_2_ (**13**) produced an ion with a considerably longer drift time (ions **bb** and **bc**, green trace) but, again, the specific hexose loss was not determined.

#### Fragments of Composition Hex_9_-O-CH=CH-O^-^ (m/z 1517)

This ion is the ^2,4^A_R-1_ fragment from the Man_9_GlcNAc_2_ glycans **9** and **10**. Extracted fragment ATDs, with different cross-sections, are shown in Figure 7b. Unlike the situation with the corresponding GlcNAc-containing ^2,4^A_R_ ion, the drift time for the ion from the isomer **10** was shorter than that of the ion from the d1d1d3 isomer **9**, The profile (blue trace) from the *S. cerevisiae*-derived glycan (**10**) was asymmetric, revealing the presence of the d1d1d3-isomer **az**. Figure 7d shows the extracted fragment ATD of *m/z* 1517 from Man_9_GlcNAc_2_ (recorded on a different occasion, hence the slightly different drift time), with that from the fragment produced by mannose loss from Glc_1_Man_9_GlcNAc_2_ (**12**, ion **bd**). Again, the ion (**az**) from the d1d1d2 isomer of Man_9_GlcNAc_2_ (**9**) had the longer drift time in contrast to the situation with the ^2,4^A_R_ ion **aw**. Ions from the Glc_3_-containing glycans were too weak to determine if significant separation occurred; most had drift times slightly less than that of ion **az**.

### Hexose_10_

**Figure 8:**
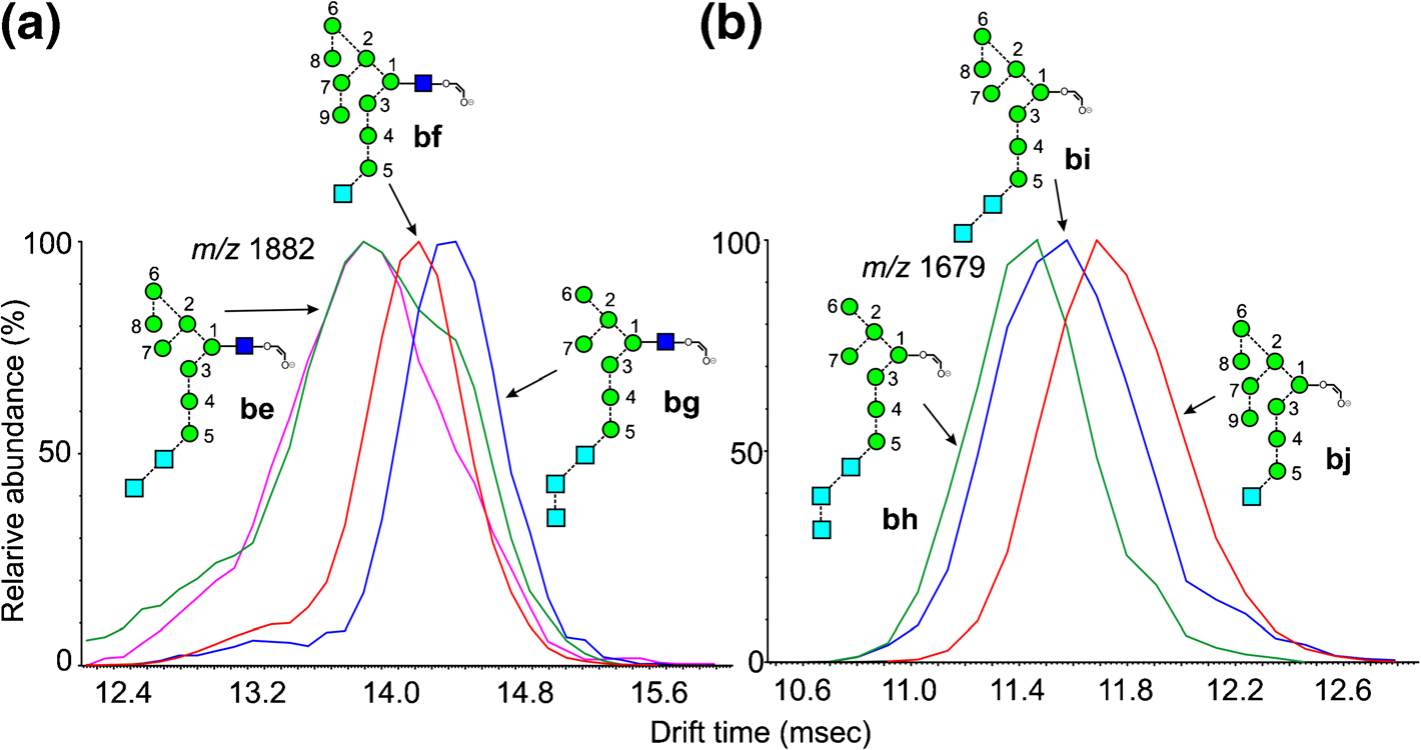
Extracted fragment ATDs of Hex_10_-containing ions. **(a)** Fragments of composition Hex_10_GlcNAc_1_-O-CH=CH-O^-^ (*m/z* 1882). Blue trace, ^2,4^A_R_ ion from Glc_3_Man_7_GlcNAc_2_ (**13**, ion **bg**). Red trace, corresponding ion (ion **bf**) from Glc_1_Man_9_GlcNAc_2_ (**12**). The green trace (smoothed) is from losses of hexose from Glc_3_Man_8_GlcNAc_2_ (**14**, ion **be**) whereas the pink trace (smoothed) is from Glc_3_Man_9_GlcNAc_2_ (**15**). (**b**) Fragments of composition Hex_10_-O-CH=CH-O^-^ (*m/z* 1697). Red and green traces, ^2,4^A_R_ ions from Glc_1_Man_9_GlxNAc_2_ (**12**) and Glc_3_Man_7_GlcNAc_2_ (**13**) respectively, Blue trace, fragments from Glc_3_Man_8_GlcNAc_2_ (**14**)

These fragments were only found in the spectra of the glucose-containing glycans and are shown in Figure 8.

#### Fragments of Composition Hex_10_GlcNAc_1_-O-CH=CH-O^-^ (m/z 1882)

The extracted fragment ATDs of this ion as the ^2,4^A_R_ fragment from Glc_1_Man_9_GlcNAc_2_ (**12**, 428.3) and Glc_3_Man_7_GlcNAc_2_ (**13**, 431.6) produced different drift times (ions **bf**, red trace and **bg**, blue trace, respectively, Figure 8a). Again, the glycan with three glucose residues in the 3-antenna produced the longer drift time, reflecting its less compact structure. Glc_3_Man_8_GlcNAc_2_ (**14**, green trace) produced two peaks, one of which had a drift time coincident with that of ion **bg** and one with a considerably shorter drift time. The ion with the longer drift time could be formed by loss of mannose 8 to give ion **bg** or by loss of a glucose residue to give **be**. Glc_3_Man_9_GlcNAc_1_ (**15**, pink trace) also gave a minor ion of undetermined structure at this drift time.

#### Fragments of Composition Hex_10_-O-CH=CH-O^-^ (m/z 1679)

The ATDs of the ^2,4^A_R-1_ ions from Glc_1_Man_9_GlcNAc_2_ (**12**, ion **bj**, red trace, CCS 390.2 Å^2^) and Glc_3_Man_7_GlcNAc_2_ (**13**, ion **bh**, green trace, 380.0 Å^2^) were well separated but, as with the corresponding Hex_9_ fragments above, their relative drift times were reversed from the ions containing GlcNAc (Figure 8b). Proposed structures are in Figure 8b. Glc_3_Man_9_GlcNAc_2_ (**15**) produced an ion with a drift time identical to that from the corresponding ion from Glc_1_Man_9_GlcNAc_2_, (**12**) presumably by loss of two glucose residues, whereas Glc_3_Man_8_GlcNAc_2_ (**14**) produced a third ion of indeterminate structure (probably **bi**).

## Conclusions

This work shows that the ATDs of fragment ions in the negative ion CID spectra of high-mannose *N*-glycans can be used to obtain additional structural and fragmentation information to that present in the CID spectra themselves. Most of the ions that provided useful information fell into two groups; those arising from the ^2,4^A_R_ fragments by additional losses from the antennae, or from corresponding fragmentation of the ^2,4^A_R-1_ ions. Many of the ions showed sufficient differences in collision cross-section to enable separation to baseline. Of particular significance is the additional information that this method provided on the presence of isomers. For example, although all three isomers of Man_8_GlcNAc_2_ can be identified individually by their negative ion CID spectra, determination of the relative amounts of, for example, the d1d1d2 isomer (**7**) in the presence of the d1d1d3 isomer (**6**) is difficult because the former isomer is only identified by the absence of the fragments at *m/z* 485 and 463. However, the CCSs of the ^2,4^A_R_ ions from these isomers are significantly different from each other and from that of the third isomer (**8**) to allow easy differentiation.

The CID spectra contained ions specific to the composition of the 6-antenna of these compounds. Specifically, an ion formed by loss of the chitobiose core and 3-antenna, known as the D ion, is of particular interest. However, in some cases, other ion structures with the same mass can sometimes occur from fragmentation of other parts of the molecules, meaning that positive identification of the D ion is sometimes difficult. In this work, it was found that the D ion at *m/z* 647 from high-mannose glycans with three mannose residues in the 6-antenna, and *m/z* 688 from biantennary glycans, produced unique CCSs allowing them to be identified. In general, glycans with mannose 8 in the 6-antenna produced longer drift times than most of the other isomers. The exception was glycans with three glucose residues capping the 3-antenna. These compounds invariably produced cross-ring fragment ions (Glc_3_Man_1_-O-CH=CH-O^-^ or Glc_3_Man_2_-O-CH=CH-O)^-^ with high CCS values. These ions are prominent in the CID spectra and are used as diagnostic fragments for this structural feature. In general, linear ions such as these appeared to have higher CCS values than isomeric ions with branched structures. Other structural features such as the presence of α-galactose residues on the non-reducing termini of the antenna also gave specific CCS values. These moieties are of interest to the biopharmaceutical industry because of their antigenic properties.

Although many of the ATDs shown above appeared to be produced by single ions, the possibility that some contained contributions from other minor fragments with similar CCSs cannot be ruled out. This situation is most likely to occur when fragmentation involves more than one additional stage. However, formation of many of the fragments could be rationalized by a single additional cleavage from the ^2,4^A_R_ or ^2,4^A_R-1_ ions, and, in these cases, the ATDs probably represented single structures. Another point to be taken into account is that the above data were recorded from a comparatively small selection of glycans, although most possible high-mannose glycans were included. Thus, CCSs, such as those from the D ions, although appearing to be unique, may not, in fact, be so. However, even in these situations, the absence of a peak with the specific CCS of these ions can be taken as positive information on their absence.

The shape of the ATD peaks also provided some information on fragmentation mechanisms. Thus, fragmentation of the larger high-mannose glycans such as Man_9_GlcNAc_2_ (9) by loss of a single α-mannose residue can involve any of the three residues present at the non-reducing termini of the three antennae. The very broad, ill-defined peak that was observed spanning the CCS range for the ^2,4^A_R_ ions from the three Man_8_GlcNAc_2_ isomers observed for this ion showed that the loss from Man_9_GlcNAc_2_ involved any of the terminal mannose residues in roughly equal amounts.

Further work in this area will focus on positive ions to ascertain if corresponding structural information can be obtained in a manner similar to recent work that provided structural information on Lewis epitopes.

## Electronic supplementary material

ESM 1 (DOCX 2959 kb)
