# Supplementary material for: Isomer Information from Ion Mobility Separation of High-Mannose Glycan Fragments
Source: J Am Soc Mass Spectrom. 2018 Mar 5;29(5):972–88. doi: 10.1007/s13361-018-1890-5 (PMC5940726; doi:10.1007/s13361-018-1890-5)
Supplement: Supplementary file 1 — (DOCX 2959 kb) [file 13361_2018_1890_MOESM1_ESM.docx]

**Isomer Information from Ion Mobility Separation of High-Mannose Glycan Fragments**

David J. Harvey, Gemma Seabright, Snezana Vasiljevic, Max Crispin and Weston B. Struwe

**Supplementary information**

Trap fragmentation spectra for the *N*-glycans reported in this paper. Annotations in blue refer to the ion structures in Table 1.


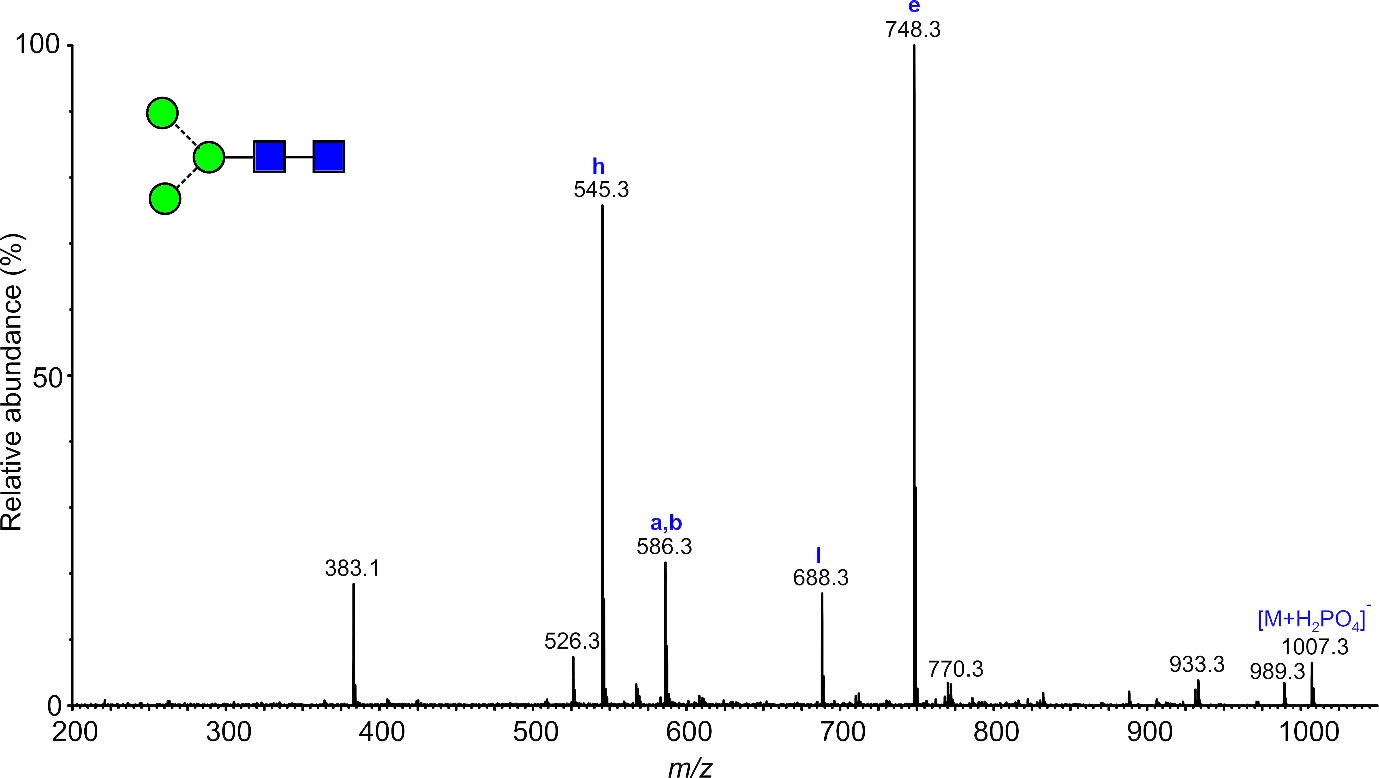


Trap fragmentation spectrum of Man_3_GlcNAc_2_ (**1**)


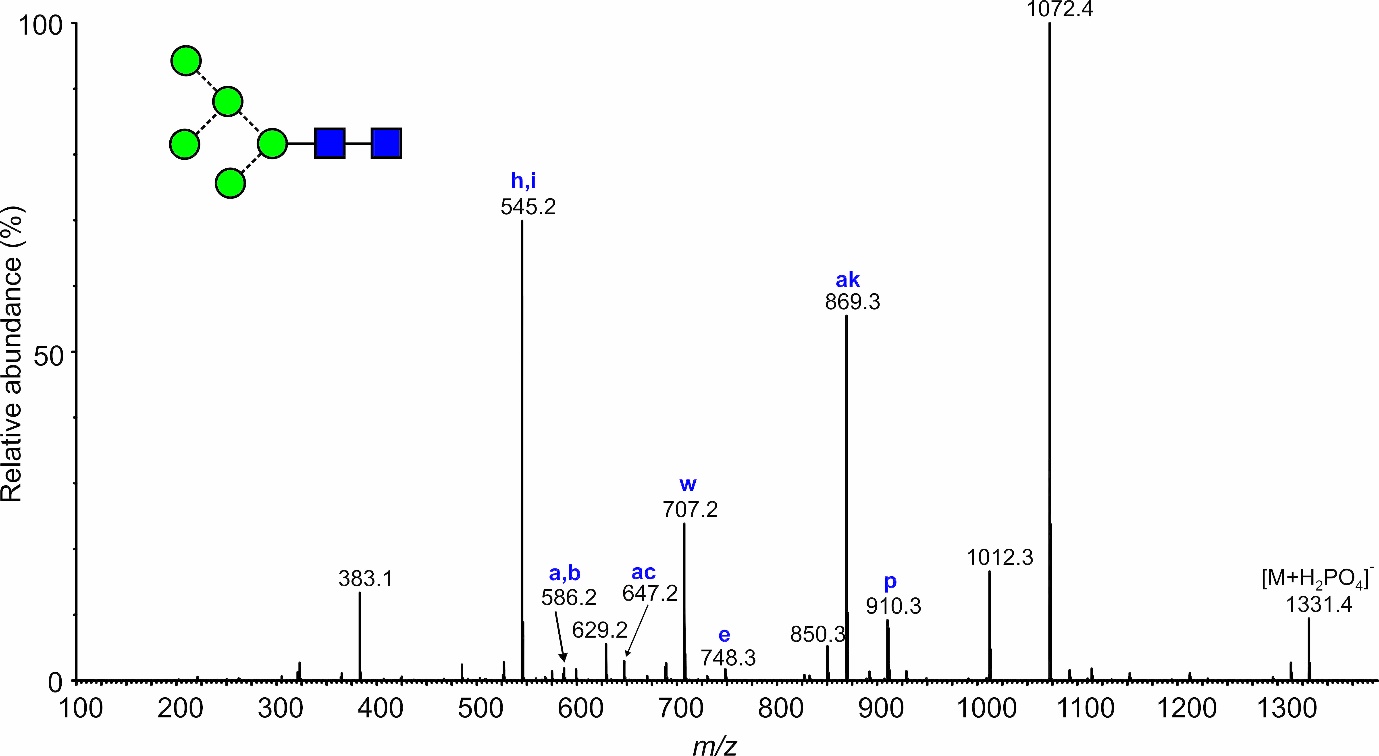


Trap fragmentation spectrum of Man_5_GlcNAc_2_ (**2**)


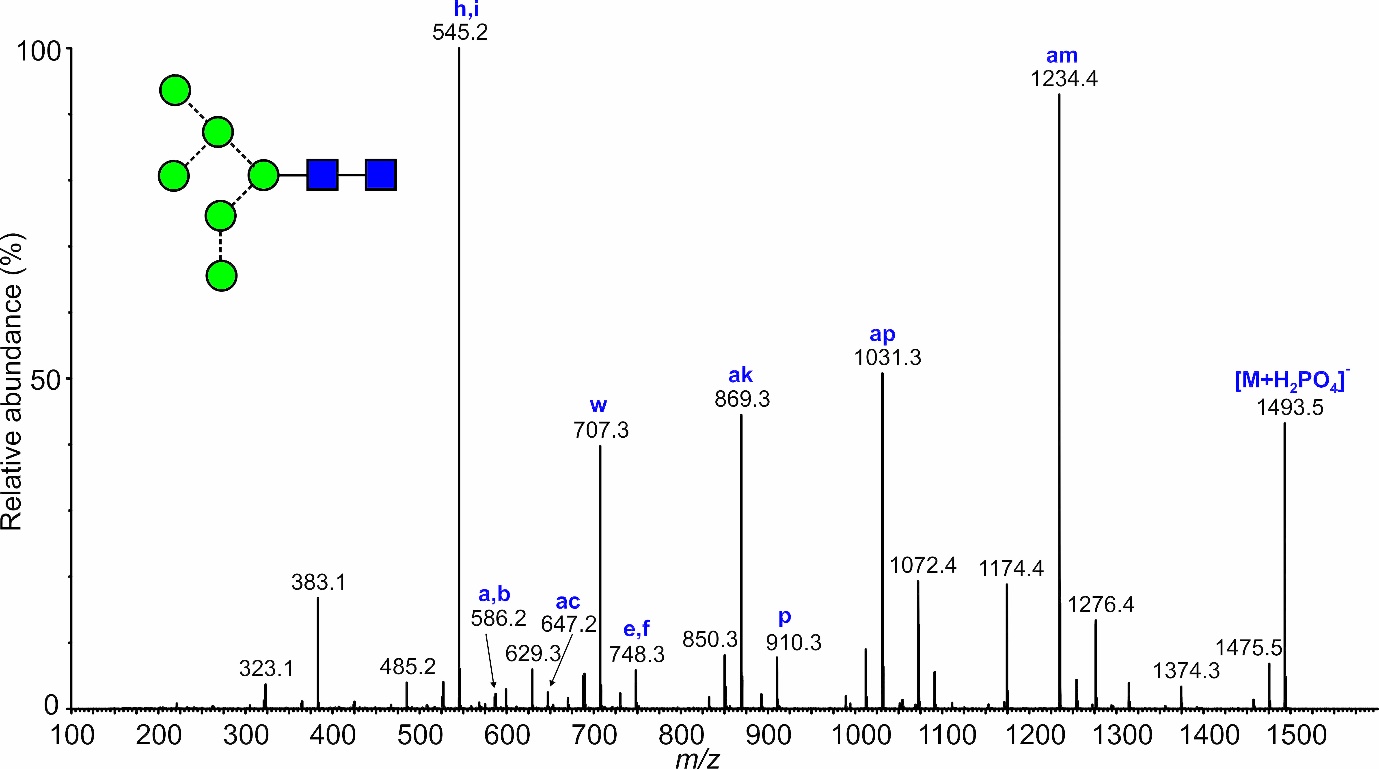


Trap fragmentation spectrum of Man_6_GlcNAc_2_ (**3**)


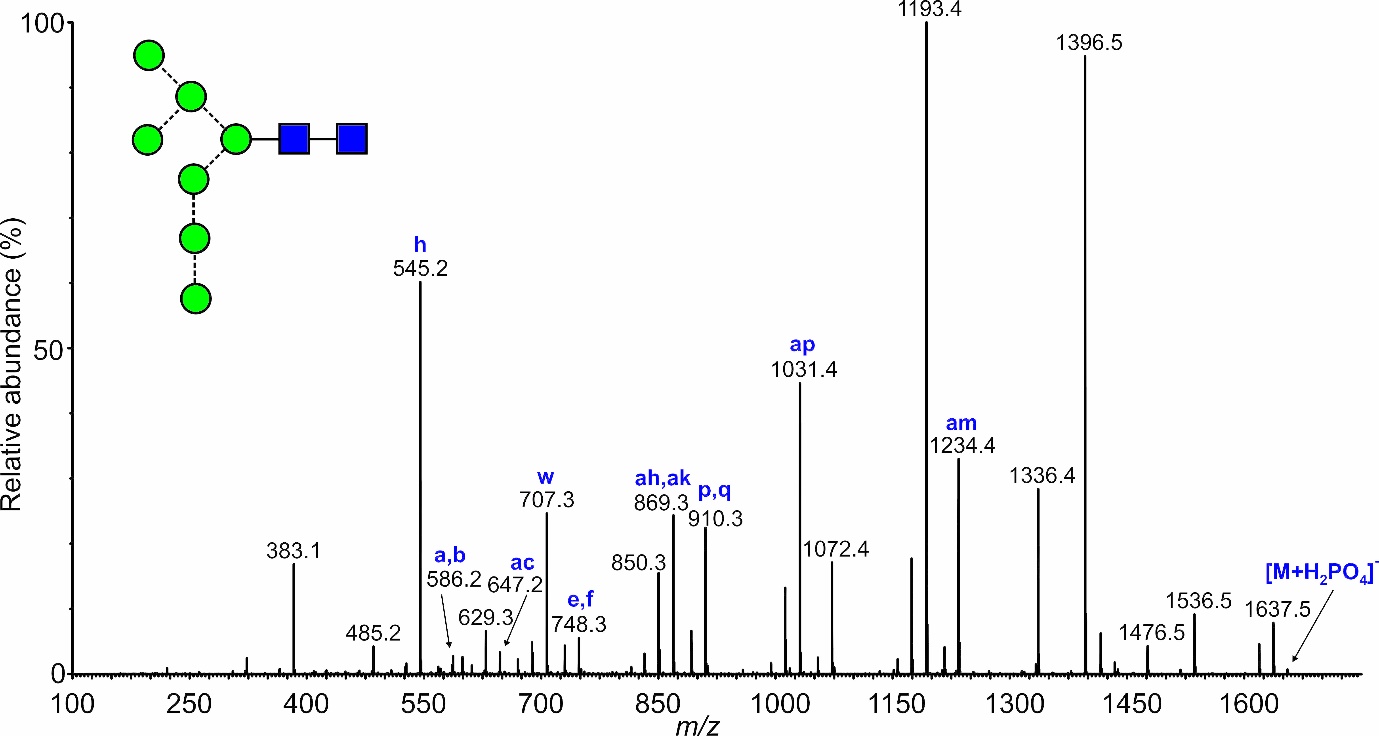


Trap fragmentation spectrum of Man_7_GlcNAc_2_ d1d1 isomer (**4**)


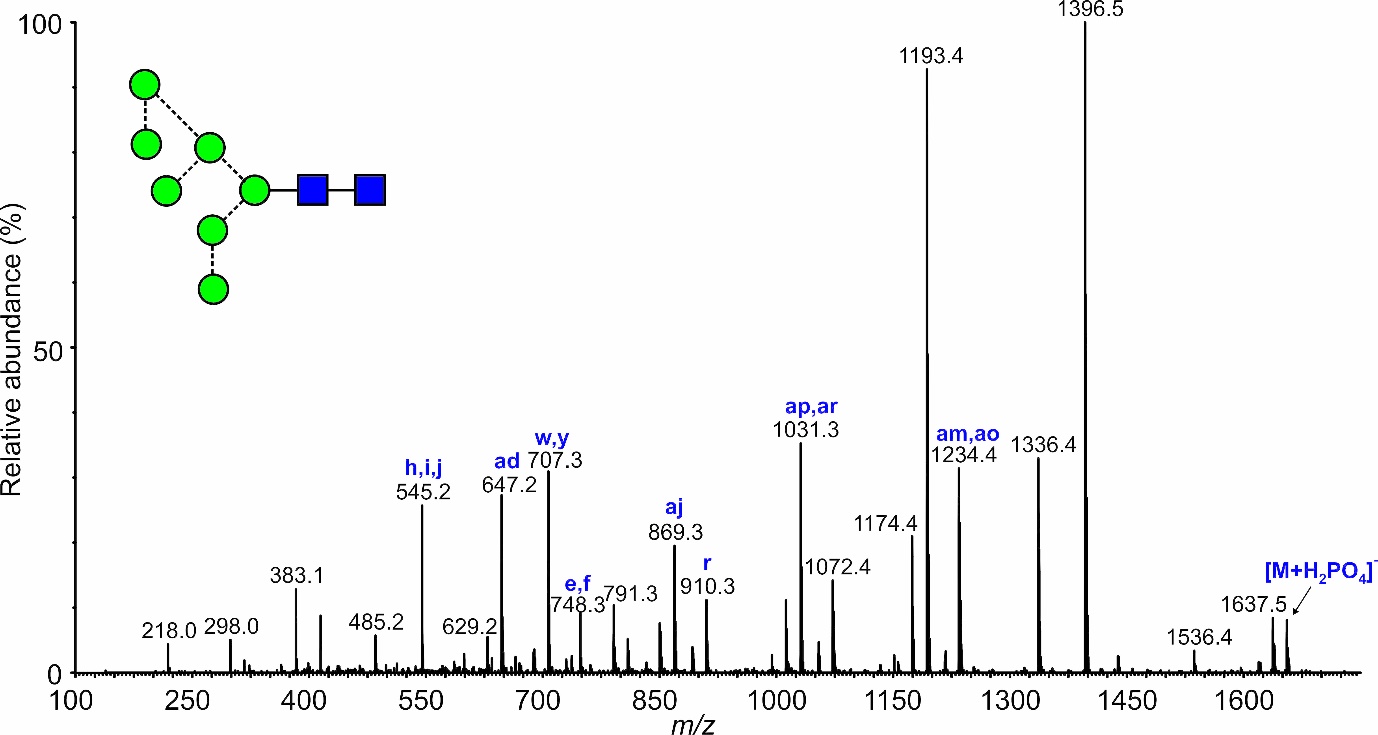


Trap fragmentation spectrum of Man_7_GlcNAc_2_ d1d3 isomer (**5**)


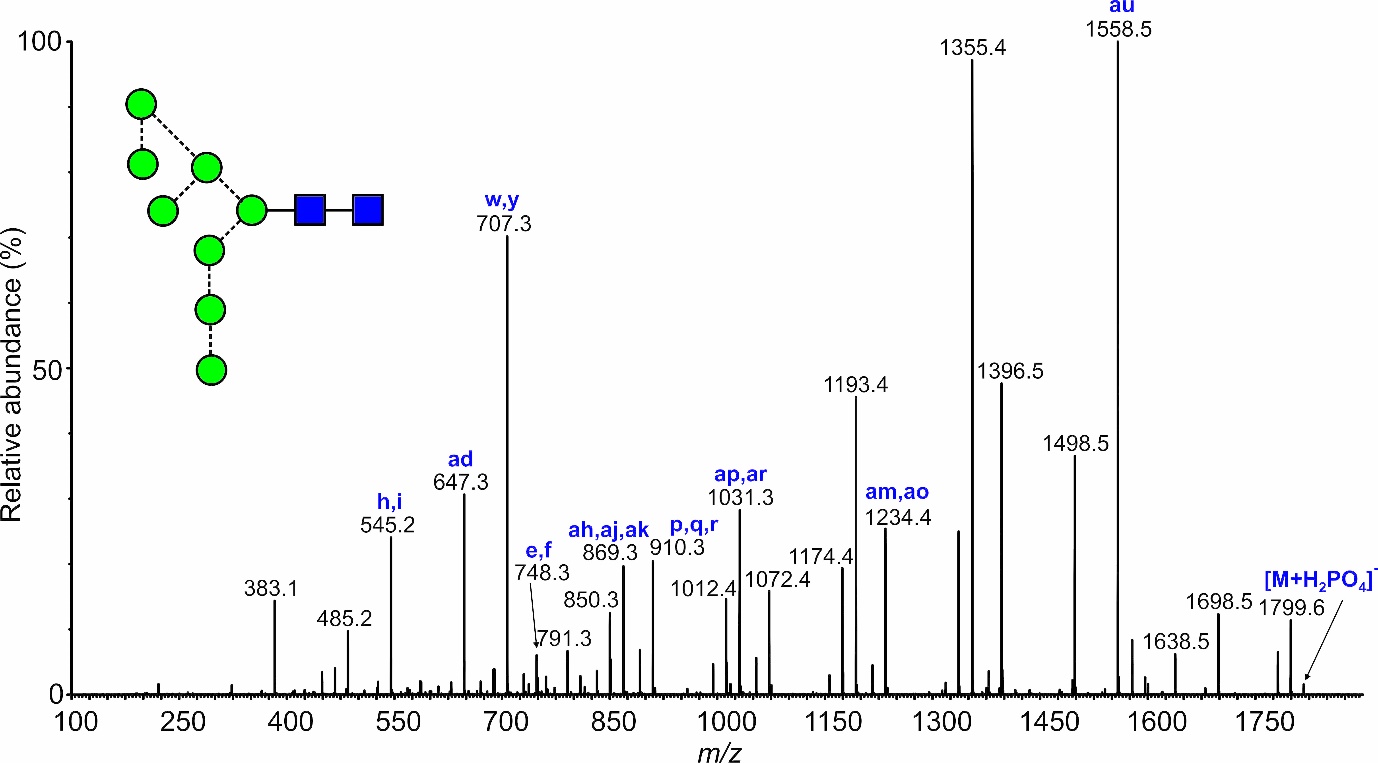


Trap fragmentation spectrum of Man_8_GlcNAc_2_ d1d1d3 isomer (**6**)


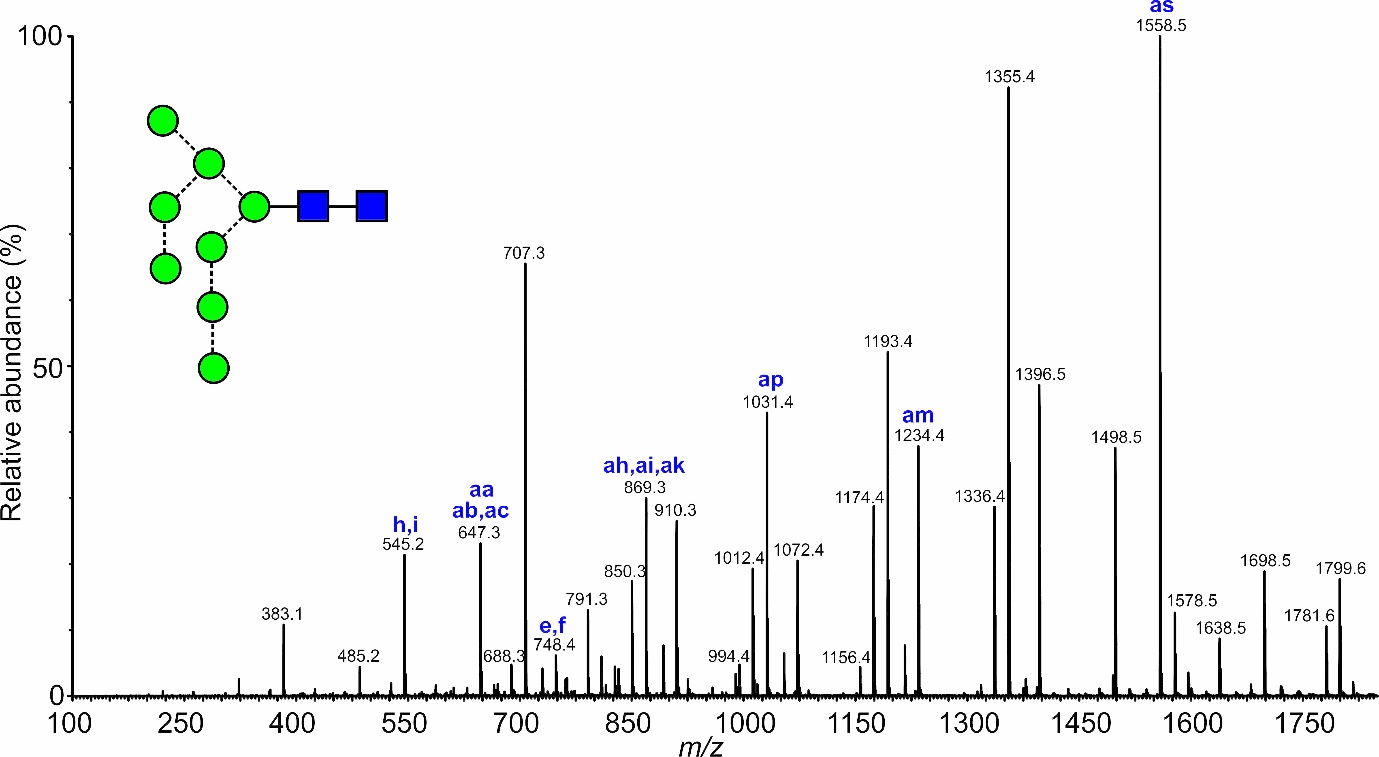


Trap fragmentation spectrum of Man_8_GlcNAc_2_ d1d1d2 isomer (**7**)


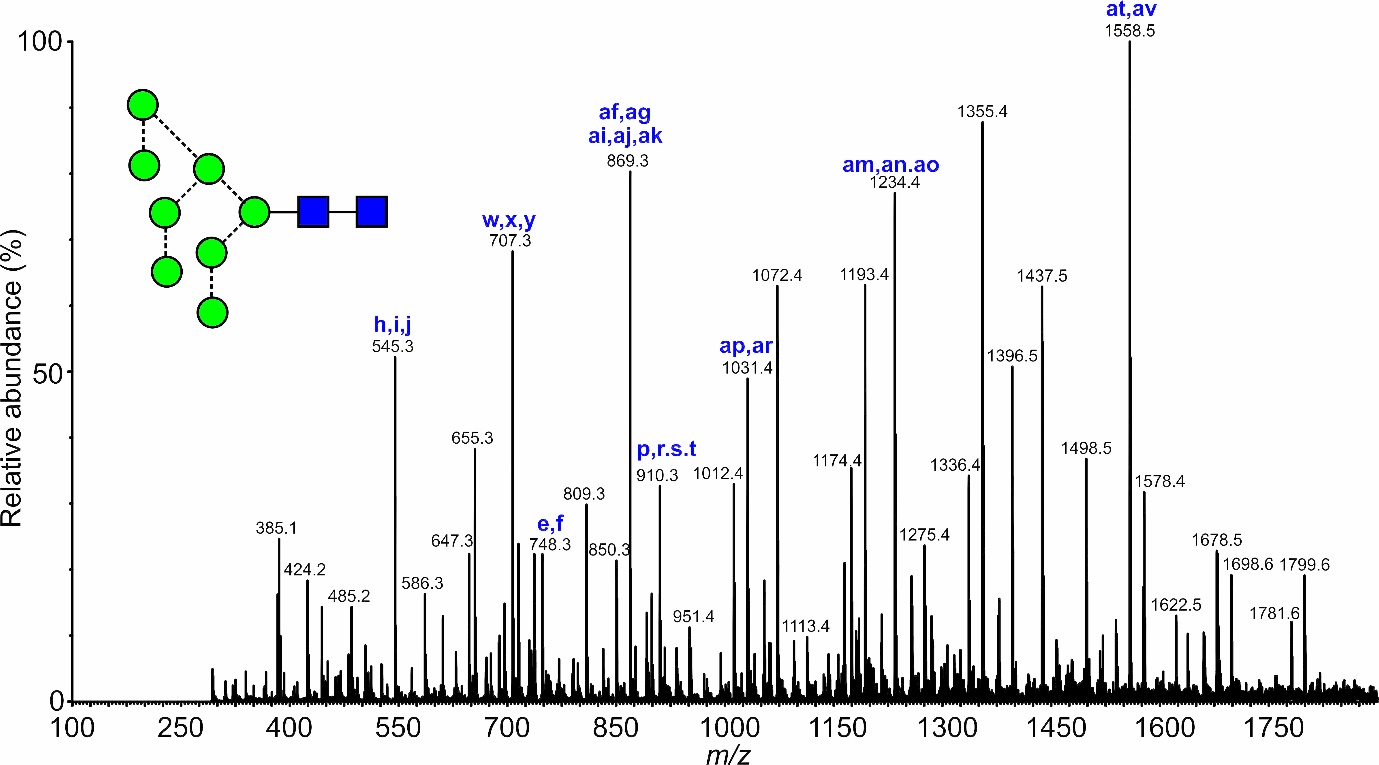


Trap fragmentation spectrum of Man_8_GlcNAc_2_ d1d2d3 isomer (**8**) (weak spectrum)


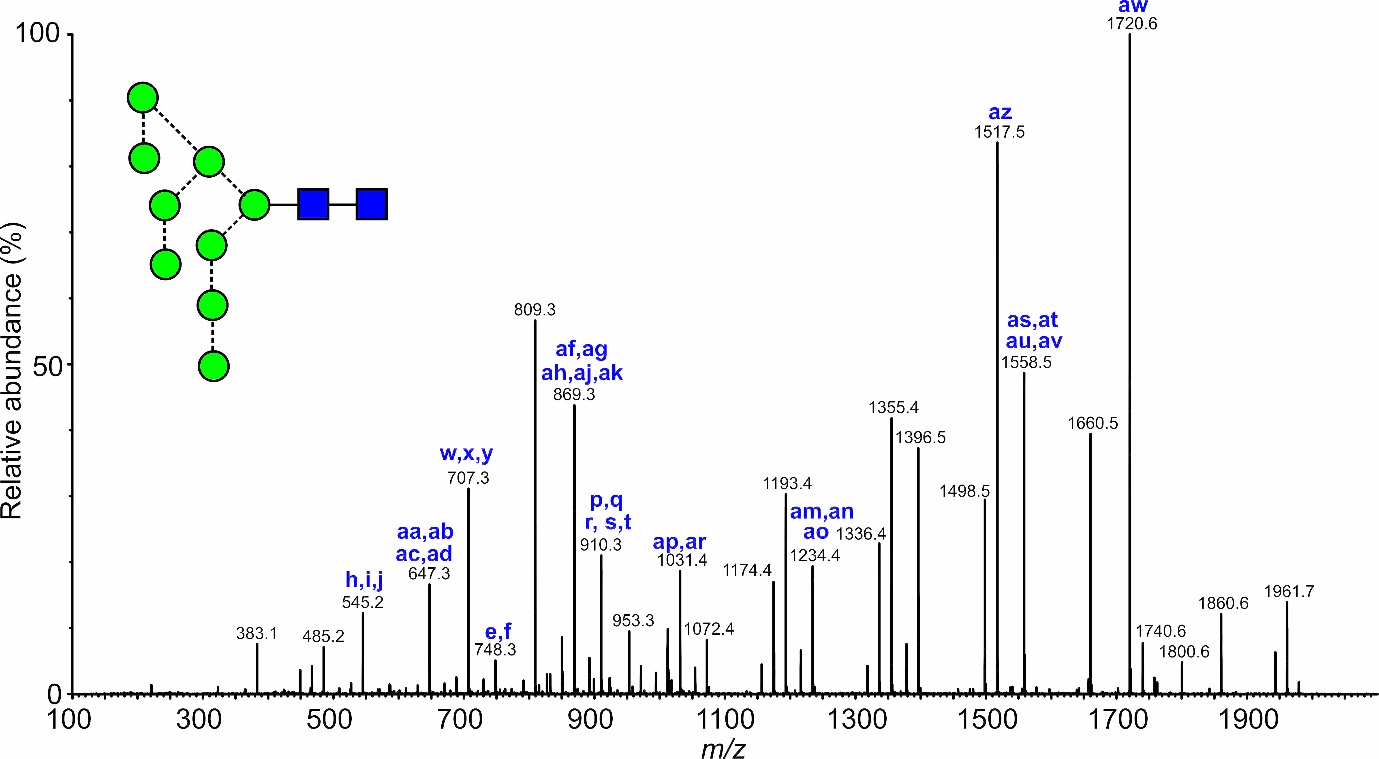


Trap fragmentation spectrum of Man_9_GlcNAc_2_ (**9**)


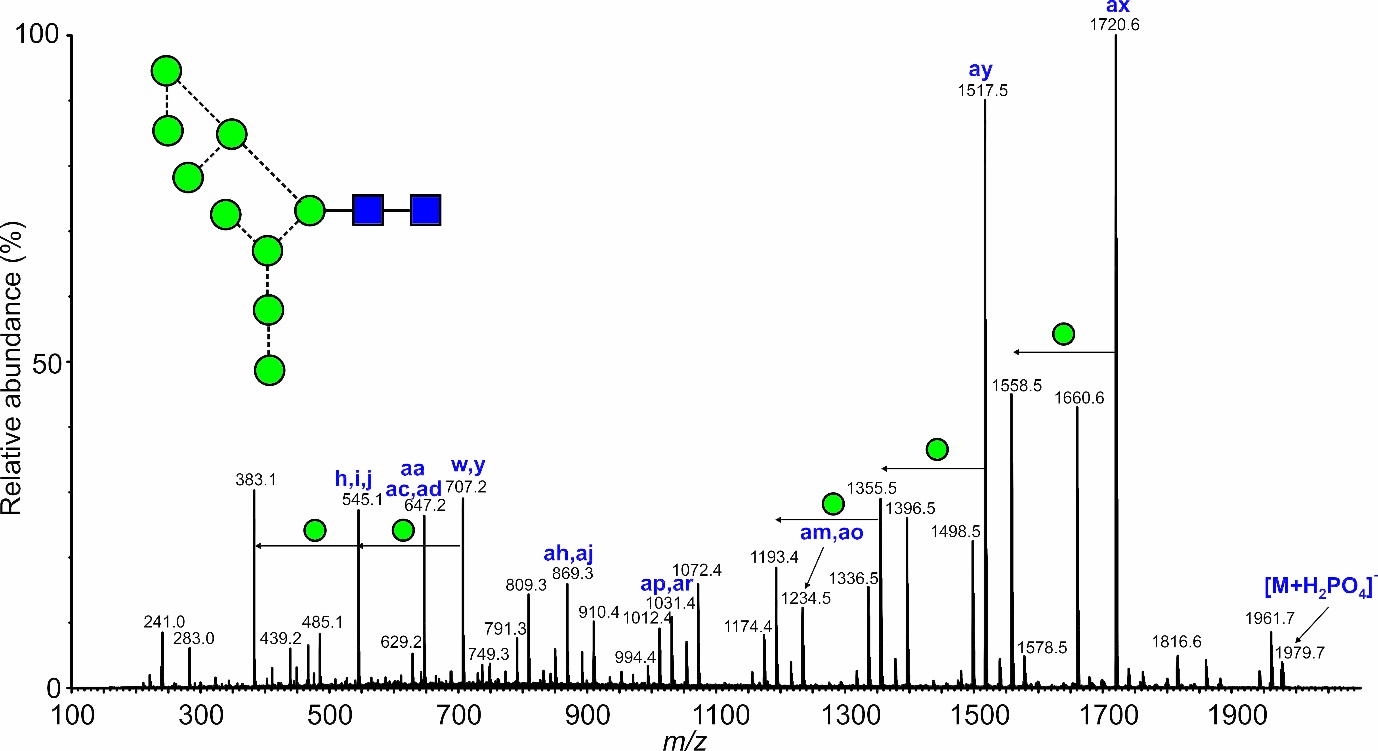


Trap fragmentation spectrum of Man_9_GlcNAc_2_ from yeast (**10**)


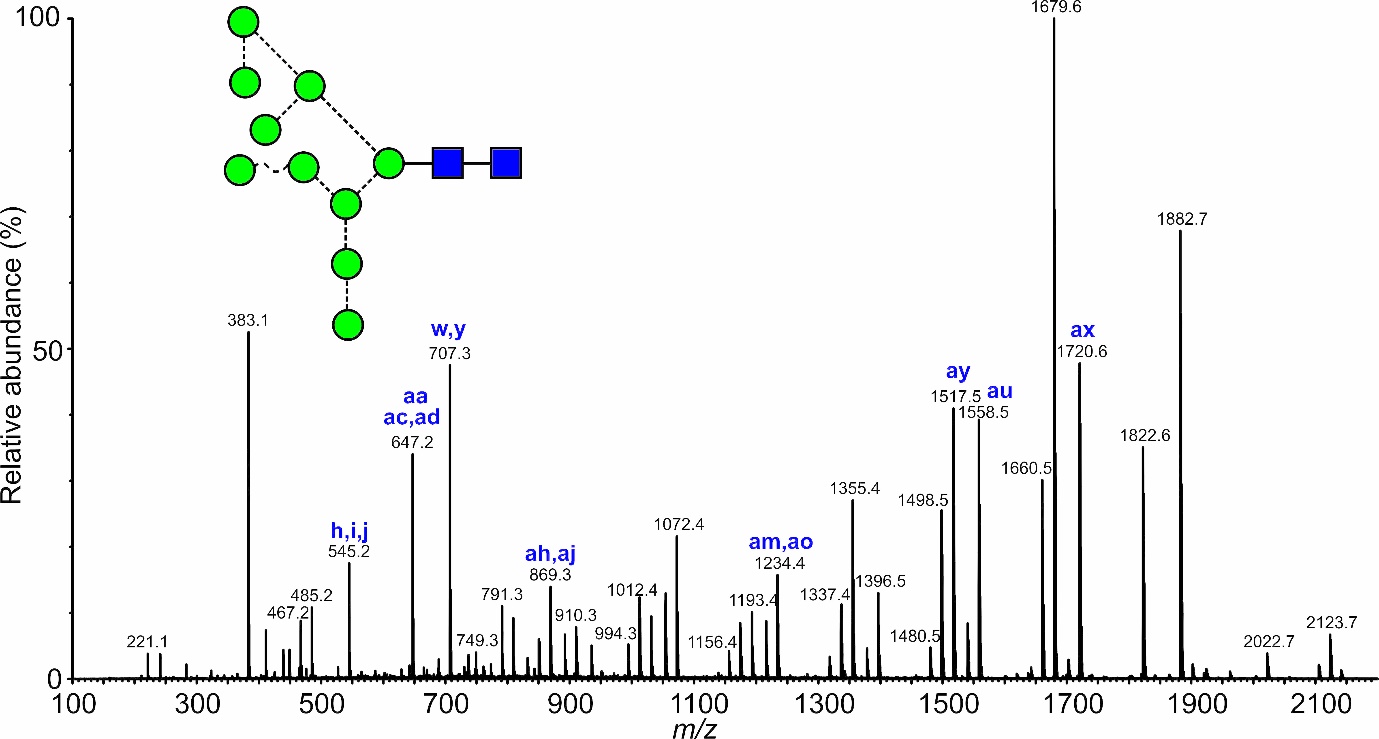


Trap fragmentation spectrum of Man_10_GlcNAc_2_ from yeast (**11**)


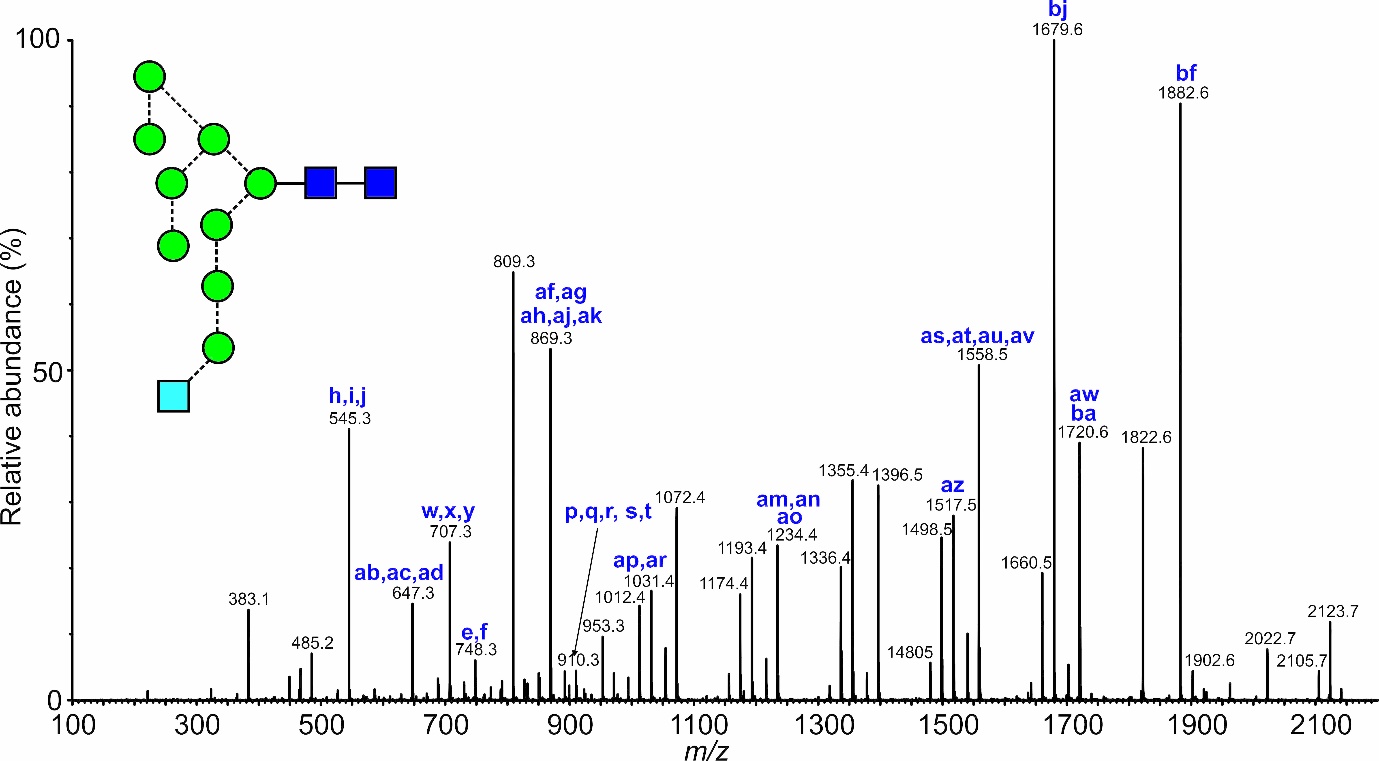


Trap fragmentation spectrum of Man_9_GlcNAc_2_Glc_1_ (**12**)


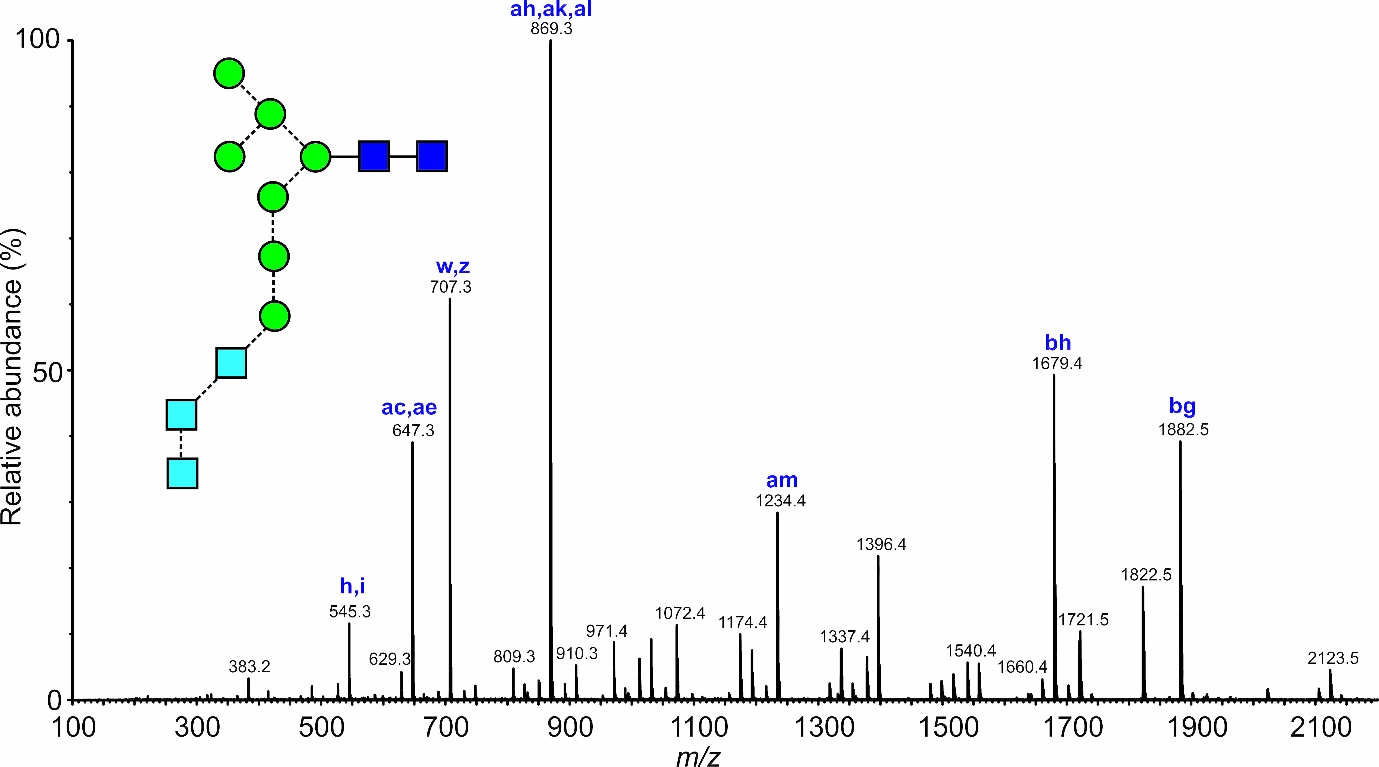


Trap fragmentation spectrum of Man_7_GlcNAc_2_Glc_3_ (**13**)


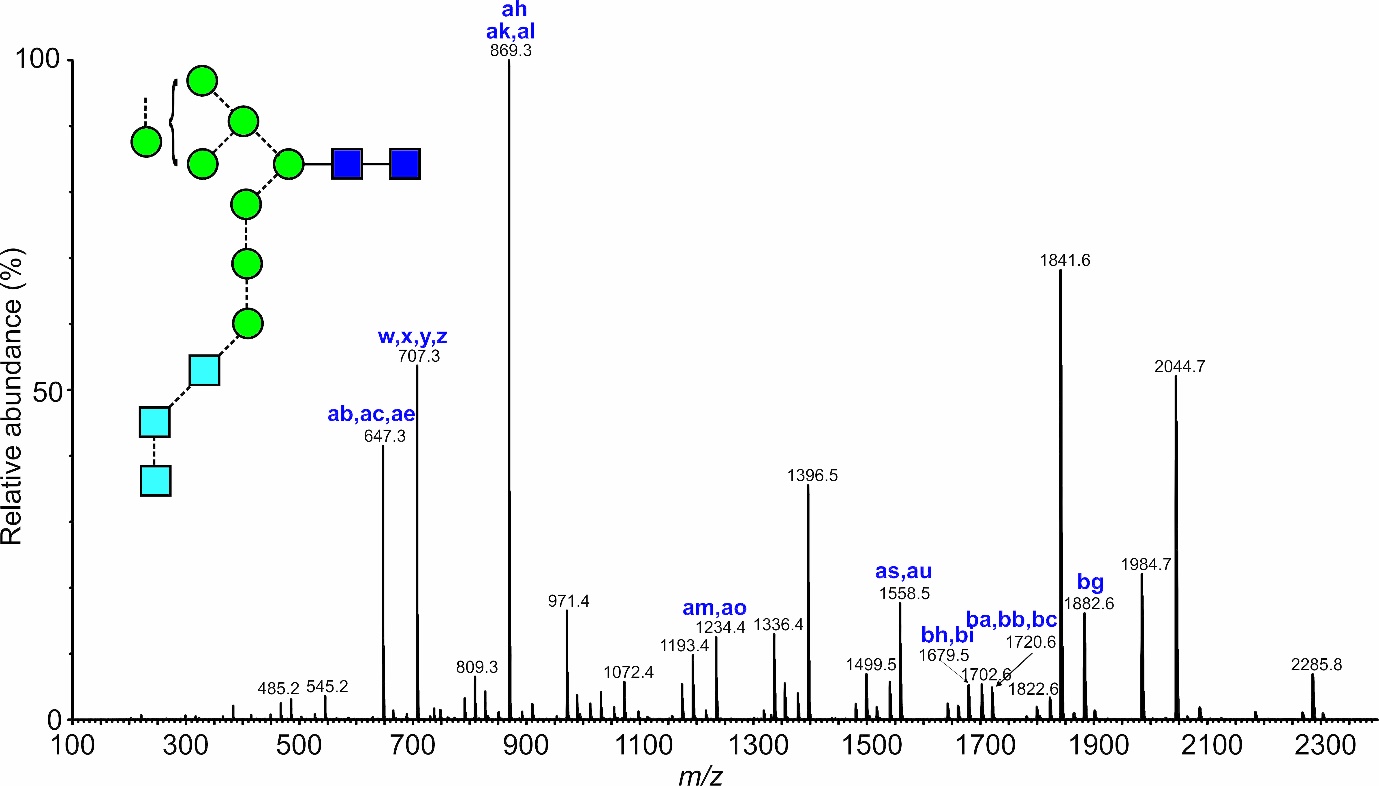


Trap fragmentation spectrum of Man_8_GlcNAc_2_Glc_3_ (**14**)


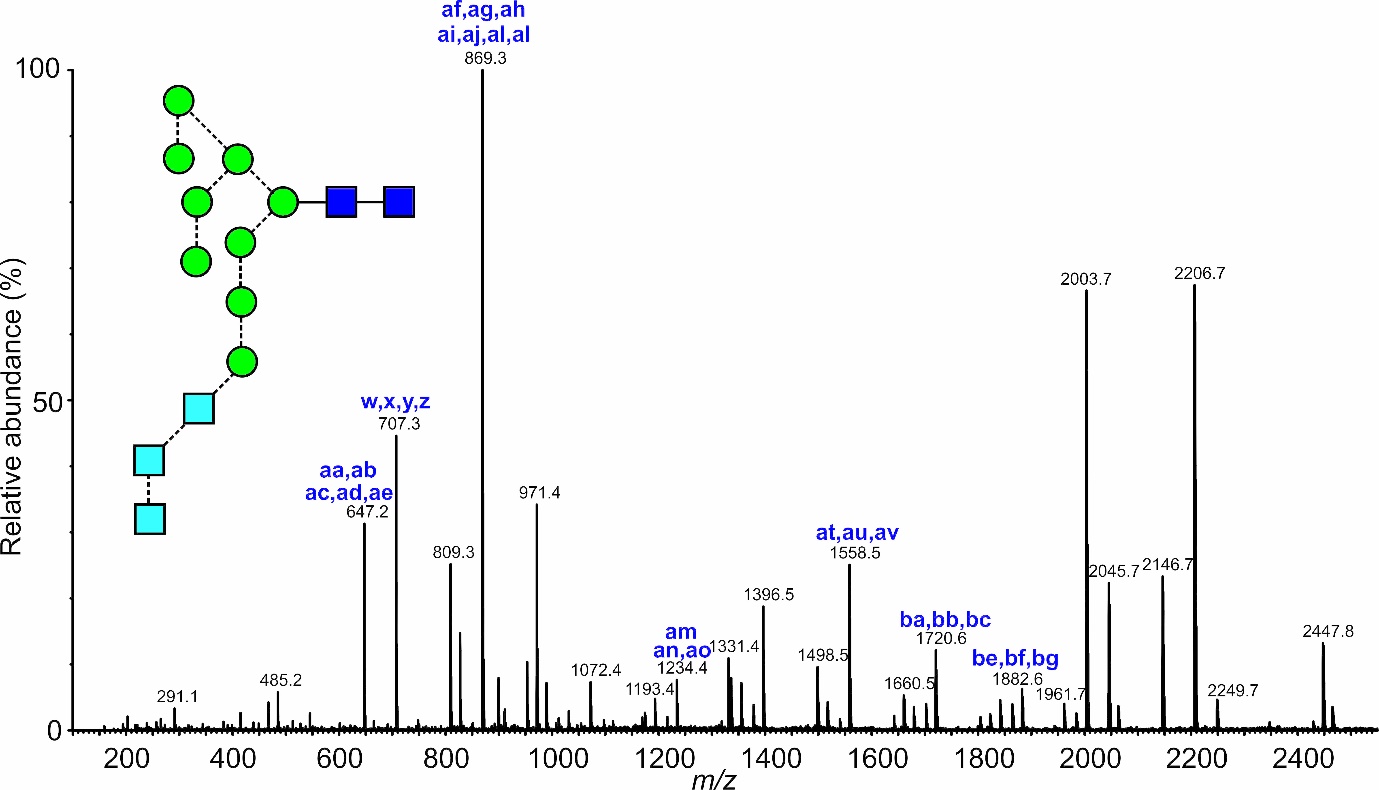


Trap fragmentation spectrum of Man_9_GlcNAc_2_Glc_3_ (**15**)


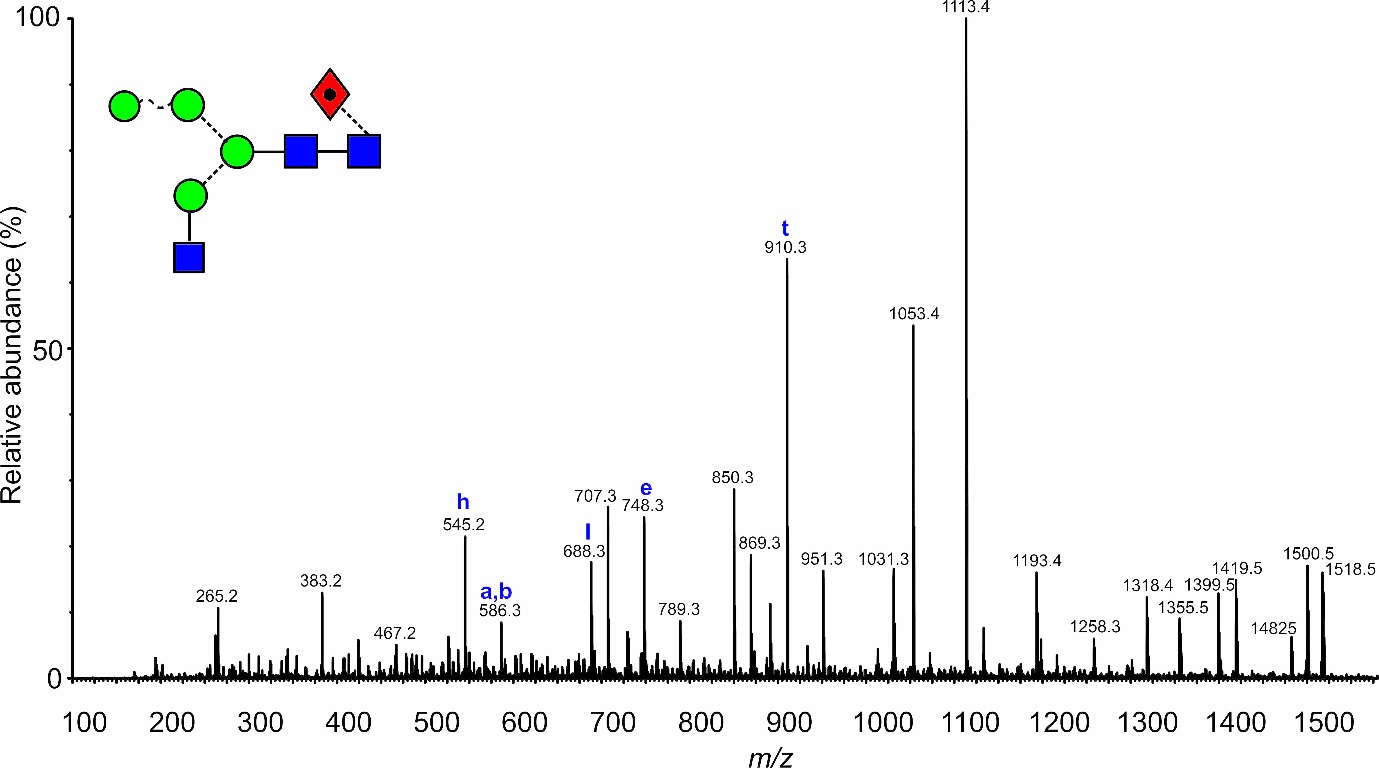


Trap fragmentation spectrum of the hybrid glycan Man_4_GlcNAc_3_Fuc_1_ (**17**)


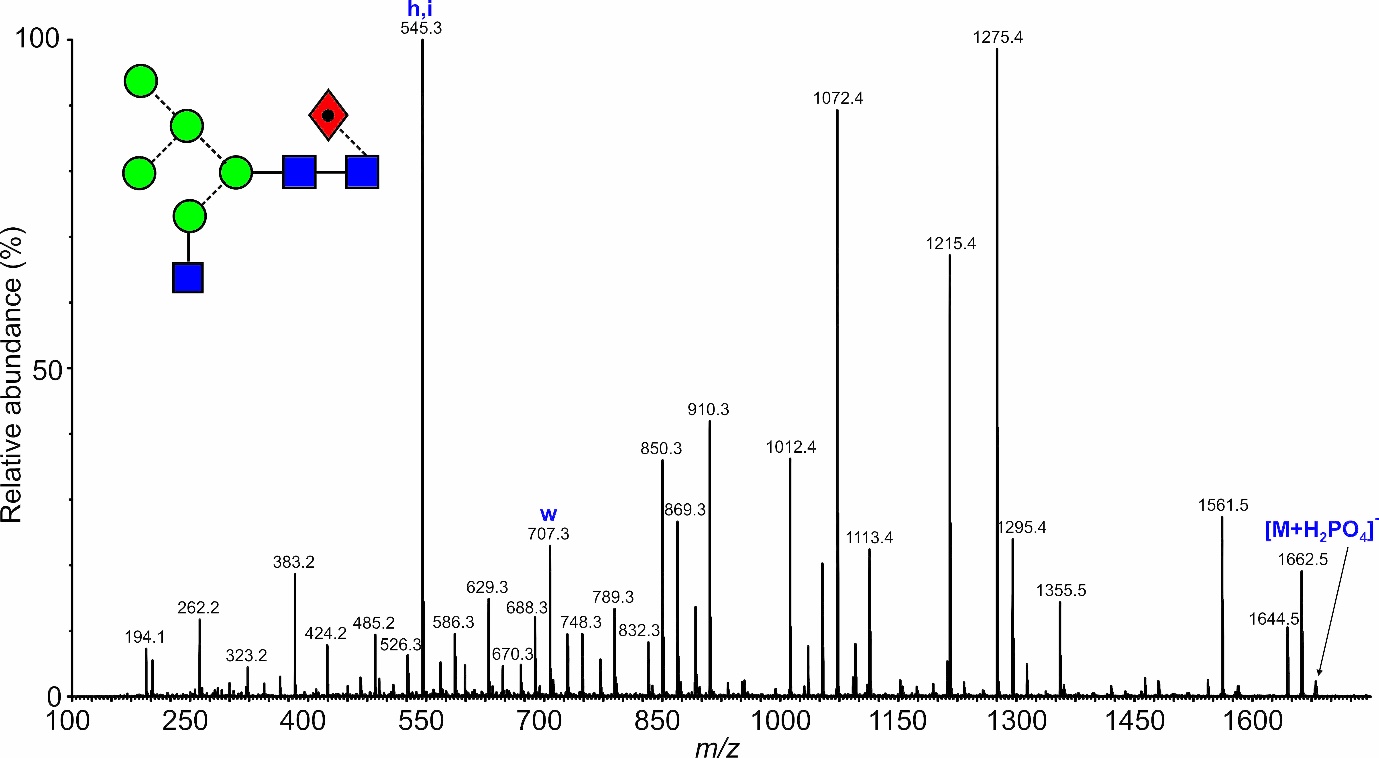


Trap fragmentation spectrum of the hybrid glycan Man_5_GlcNAc_3_Fuc_1_ (**19**)


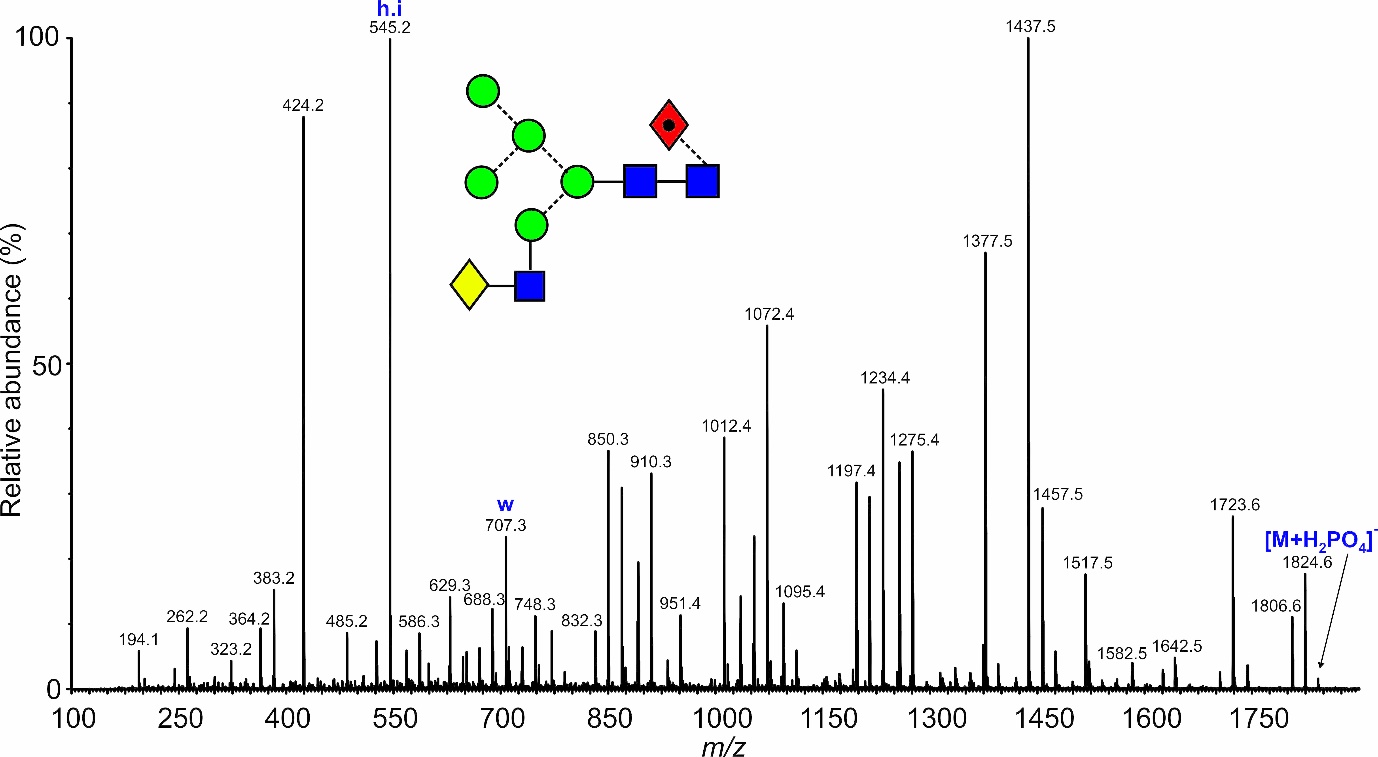


Trap fragmentation spectrum of the hybrid glycan Man_5_GlcNAc_3_Gal_1_Fuc_1_ (**20**)


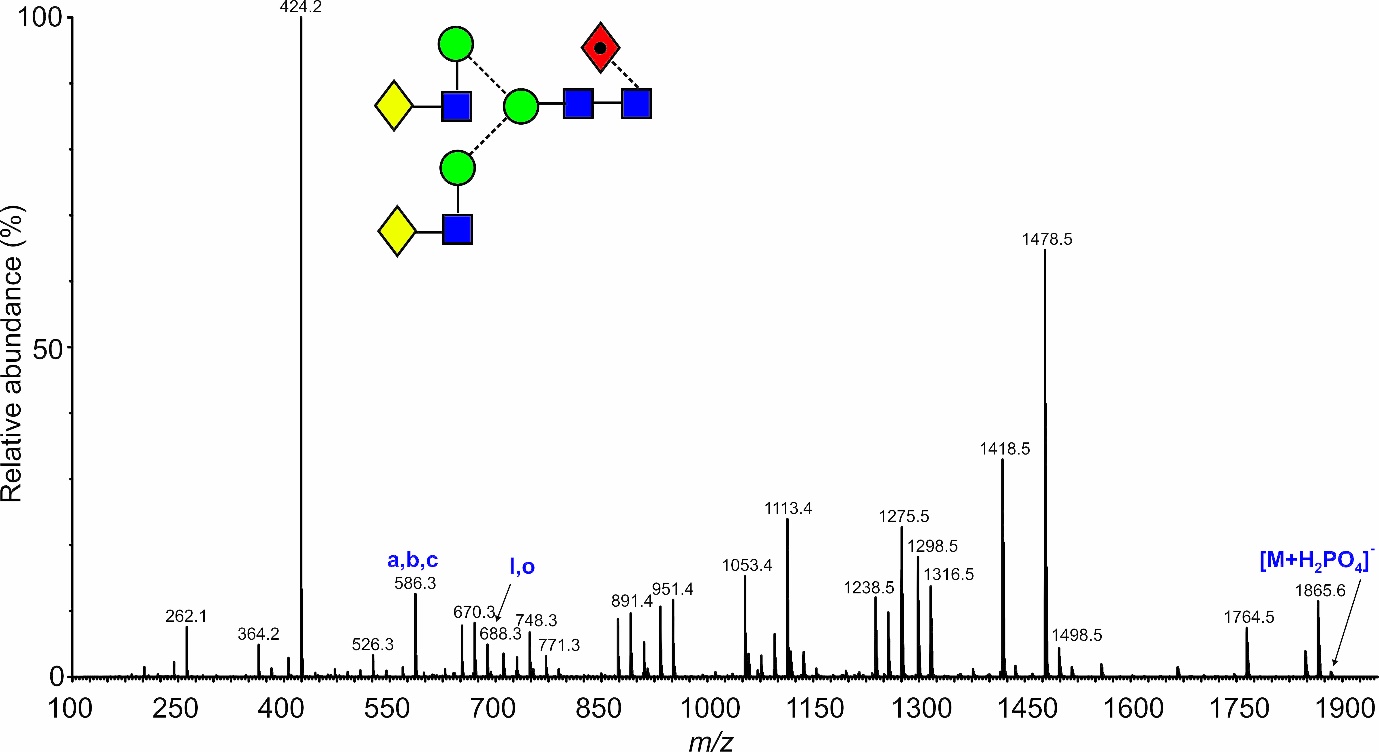


Trap fragmentation spectrum of the biantennary glycan Man_3_GlcNAc_4_Gal_2_Fuc_1_ (**21**)


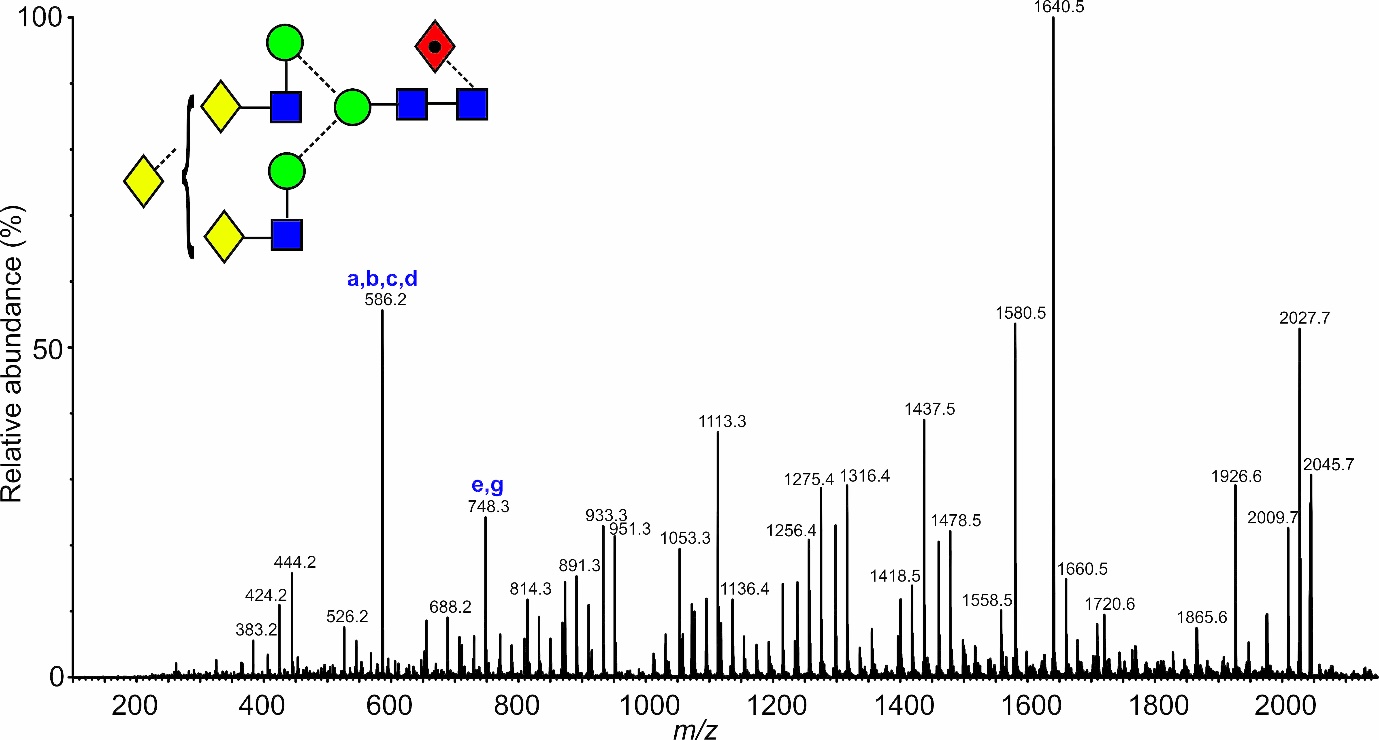


Trap fragmentation spectrum of the biantennary glycan Man_3_GlcNAc_4_Gal_3_Fuc_1_ (**22**)


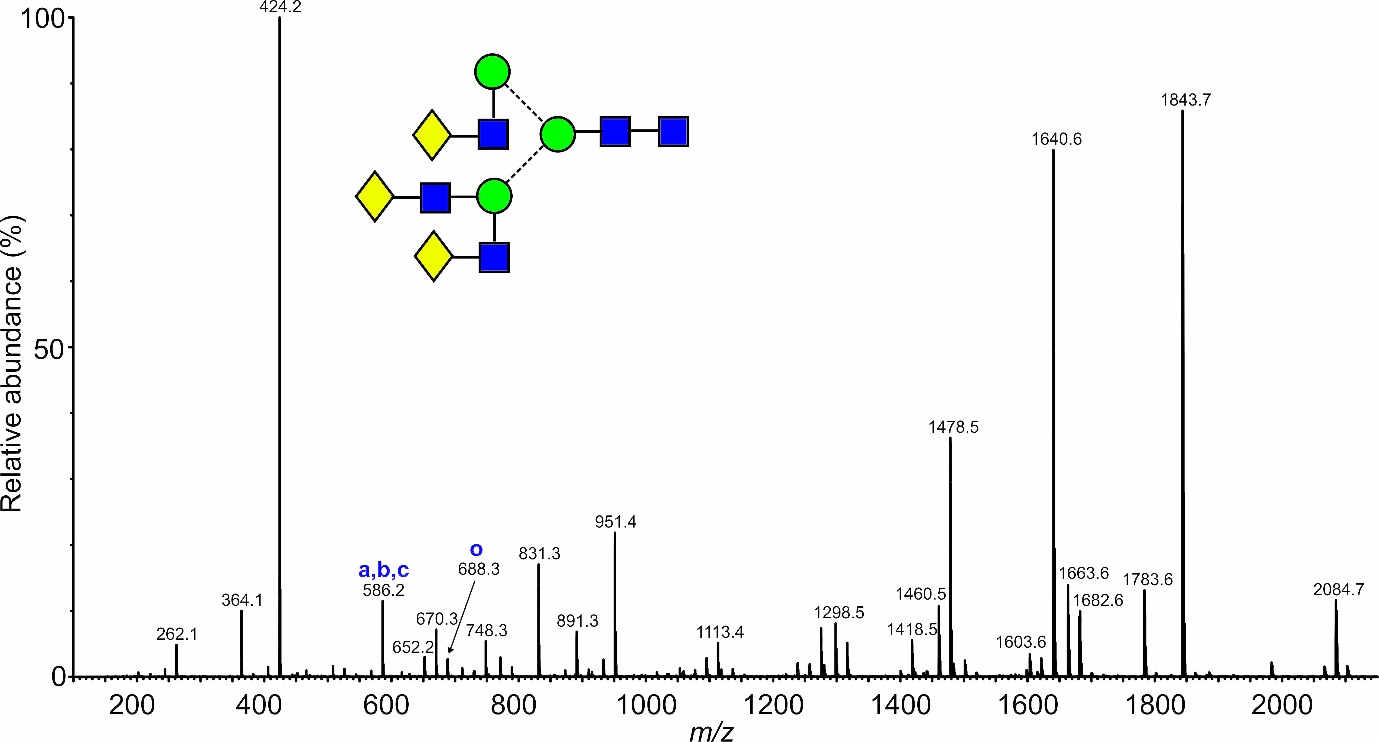


Trap fragmentation spectrum of the triantennary glycan Man_3_GlcNAc_5_Gal_3_Fuc_1_ (**23**)


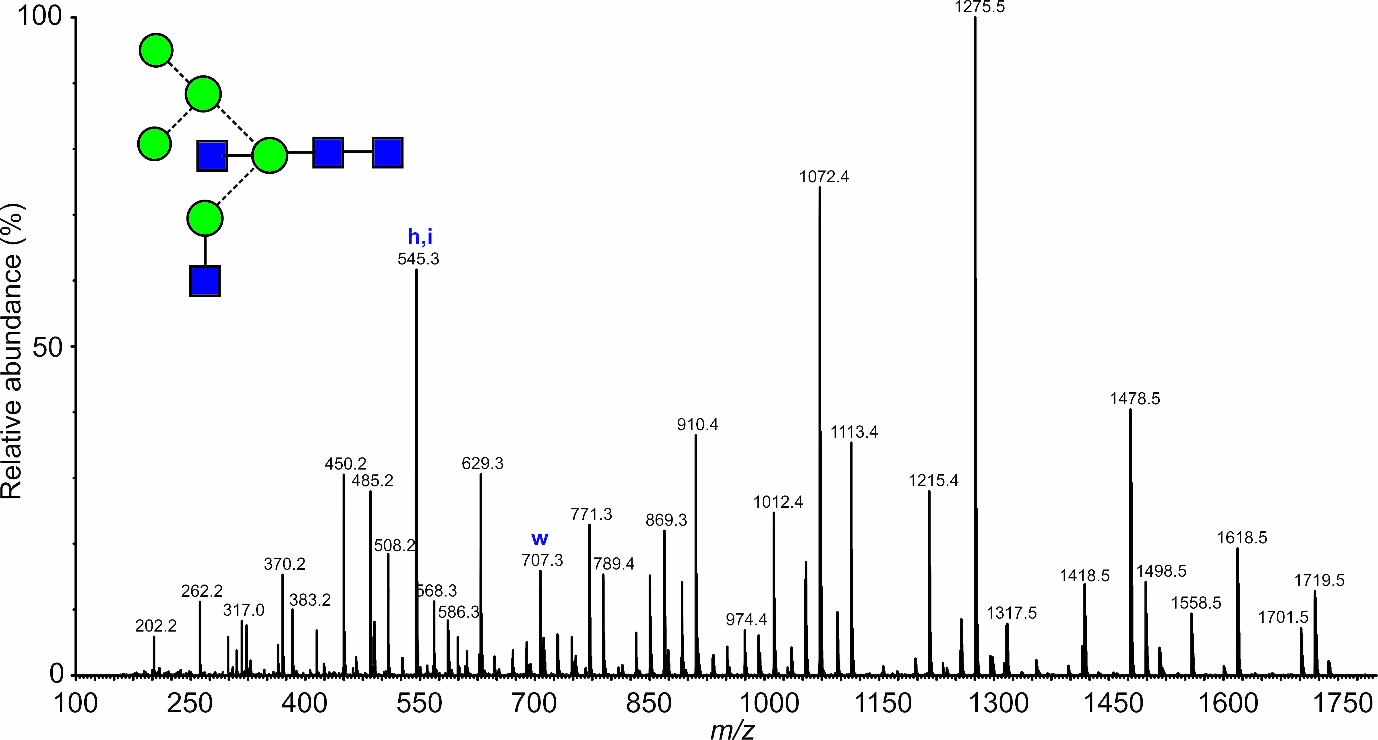


Trap fragmentation spectrum of the bisected glycan Man_3_GlcNAc_4_ (**24**)
